# Health inequalities in Brazilian adolescents: Measuring and mapping gaps in a cross‐sectional school‐based survey

**DOI:** 10.1002/hsr2.1761

**Published:** 2023-12-15

**Authors:** Andrea Wendt, Adriana K. F. Machado, Caroline S. Costa, Daniela Rachadel, Inacio Crochemore‐Silva, Javier Brazo‐Sayavera, Paula K. Hembecker, Luiza I. C. Ricardo

**Affiliations:** ^1^ Graduate Program in Health Technology Pontifícia Universidade Católica do Paraná, Graduate Curitiba Brazil; ^2^ Postgraduate Program in Epidemiology Federal University of Pelotas Pelotas Brazil; ^3^ Postgraduate Program in Physical Education Federal University of Pelotas Pelotas Brazil; ^4^ Department of Sports and Computer Science Universidad Pablo de Olavide Seville Spain; ^5^ Medical Research Council Epidemiology Unit University of Cambridge Cambridge UK

**Keywords:** adolescents, cross‐sectional study, disparities, health inequalities

## Abstract

**Background and Aims:**

This study aims to describe inequalities in health indicators according to gender, area of residence, and socioeconomic position among Brazilian adolescents.

**Methods:**

Cross‐sectional study using data from a school‐based survey carried out in Brazil in 2019. Twelve health outcomes were evaluated. Dimensions of inequality assessed were gender, area of residence, wealth and subnational region.

**Results:**

This study comprises a sample of 124,898 adolescents. The most prevalent outcome was physical inactivity (71.9%) followed by thinking life is worthless (52.6%) and bullying (51.8%). Gender inequalities were more marked for physical inactivity and thinking life is worthless with girls presenting a prevalence more than 20 p.p. higher than boys. In zero‐dose HPV, however, the prevalence in girls was 17.7 p.p. lower than in boys. Area of residence and wealth inequalities were smaller than gender disparities. Context presented a relevant role in inequality with analysis stratified by states of the country, revealing high variability in estimates.

**Conclusions:**

We highlight the need for attention to disparities between subgroups of the adolescent population, especially for gender inequalities that were the most marked for this age group.

## 1 INTRODUCTION

Adolescence is a key period for health as it is a time of significant physical, psychological, and social changes.^1^ Many habits acquired during this period can continue into adulthood, highlighting the importance of promoting healthy adolescent behaviors.^2^ However, unhealthy lifestyles are increasingly prevalent among adolescents, leading to negative consequences on their health over time.^3^, ^4^ Unhealthy behaviors such as insufficient physical activity, prolonged screen time use,^5^ unhealthy diet, alcohol and tobacco consumption,^6^, ^7^ sleeping problems,^8^ drug use,^9^ and unsafe sex^10^ are all associated with adverse health outcomes. As some of these behaviors are interrelated and could drive further psychosocial problems,^11^ studying them from a holistic perspective might be relevant, attempting to understand adolescents' behaviors as a whole. An unhealthy lifestyle during adolescence might lead to future public health problems. Therefore, tracking the prevalence of these behaviors could benefit the implementation of preventive policies.

Several social factors, including socioeconomic status, gender, or geographical area of residence, might influence health behaviors during adolescence.^12^ Recent findings emphasize that adolescence is a period shaped by the environment, and social determinant approaches are essential to understand adolescents' health and well‐being, which can be driven by the role of changing social environments to improve individuals' behaviors.^2^

Social determinants of health are even more closely related to youth's health behaviors in contexts of high inequality levels, which is the case of several global South countries, where wide discrepancies exist in distribution and access to health.^13^ In Brazil, one of the most unequal countries of the globe,^14^ evidence from 3 million people showed higher multimorbidity and mortality rates for individuals with black ethnicity, lower educational attainment, and welfare recipients.^15^ Brazilian adolescents from socially disadvantaged population groups face more violence, bullying, drug consumption, unsafe sex, poor diets, and tobacco use.^16^ However, the literature on health inequalities focusing on global South adolescents is still limited.

Thus, given the importance of socioeconomic inequalities on adolescents' health, this study aims to describe inequalities in several health indicators according to gender, area of residence, and socioeconomic position among Brazilian adolescents, as well as identify which health indicators present the widest gaps.

## 2 METHODS

### 2.1 Study design and participants

This is a cross‐sectional study with data from the latest (fourth) edition of the National Survey of School Health (Pesquisa Nacional de Saúde do Escolar—PeNSE), a school‐based survey carried out in Brazil in 2019 by the Brazilian Institute of Geography and Statistics. The sample includes 13‐ to 17‐year‐old adolescents from public and private schools, which is representative of the Brazilian territory, geographic regions, states, and capitals municipalities. PeNSE aims to monitor the risk and protective factors for the health of Brazilian adolescent students and constitutes an important source of information on adolescent health to support public policies.

### 2.2 Sampling and data collection

Sampling was performed in two stages. The first selection stage corresponded to schools, which were selected with probabilities proportional to size, and the second to the classes of students. The selection of classes depended on the size of the school and focused on those classes that covered most students aged 13−17 years, the target population of the survey. Sample size calculation was based on a prevalence of 50% and a variation coefficient of 4%.^17^ The sample was dimensioned to estimate indicators for the different geographic levels, and the sample size was calculated considering a simple random sampling in each sizing stratum. All students from the selected classes present on the day of data collection were automatically invited to respond to the survey questionnaire.

The PeNSE's instrument has been used since 2009 in Brazilian reports and is rooted in the Global School‐based Student Health Survey/World Health Organization.^17^ In 2019, the instrument was re‐evaluated to enhance comprehension of the questions, reduce response time, and revise terms that exhibited high variability across the country.^17^

Data were collected from April to September 2019 using a smartphone where structured self‐reported questionnaires were inserted. Survey modules included themes related to diet, physical activity, smoking, alcoholic beverages, other drugs, situations at home and school, mental health, sexual and reproductive health, hygiene and oral health, safety, use of health services, and body image. For the current study, we used information from all modules except those related to the use of health services.

The students who registered their agreement with the Free and Informed Consent Term participated in the research. The National Commission of Ethics in Research and the National Council of Health approved the survey (Conep n. 3.249.268, de 08.04.2019).

### 2.3 Health indicators

We proposed a set of 12 simple health indicators collected in survey self‐administrated questionnaire, as follows: physical inactivity; thinking life is worthless; bullying; having been drunk at least once; no handwashing; physical violence; smoke experimentation; zero‐dose HPV immunization; dental pain; consumption of ultra‐processed foods; drug experimentation; eating disorders.

For physical inactivity, participants were asked about frequency and duration of activities in three domains (leisure, transportation and physical education classes). Individuals who did not reach the World Health Organization recommendation (WHO) for adolescents (300 min/week)^18^ were considered inactive.

Thinking life is worthless was obtained through a single question considering the last 30 days. Participants who reported this feeling at least once in the evaluated period were classified as thinking life is worthless.

Regarding bullying, adolescents were asked about being intimidated to the level of feeling bothered, hurt, annoyed, offended, or humiliated, being ignored by colleagues, or physically hurt by colleagues in the last 30 days. Those who reported suffering any of these situations at least once in the assessed period were classified as having been bullied.

Having been drunk at least once in life, smoke experimentation, HPV vaccination, dental pain (not considering braces) in the last 6 months, and drug experimentation (weed, skunk, cocaine, crack, ecstasy, oxy, or MD, sniffed glue, popper) were estimated using a single question with answer options yes/no.

No handwashing was measured by a single question asking about the frequency of handwashing after using the toilet. Adolescents who answered any other frequency than ‘always’ were classified as no handwashing.

Physical violence indicator was calculated considering one question regarding physical aggression by parents/caregiver and one question about aggression by someone else in the last 12 months. Adolescents who answered positively for either question were classified as having suffered physical violence.

We considered adolescents having eating disorders if they answered positively to having vomited, taken laxatives or medication to lose weight without prescription, or avoided gaining weight in the last 30 days.

To evaluate the consumption of ultra‐processed foods (UPF), we calculated a score by summing up the positive answers to a set of questions about consuming 13 ultra‐processed food items or groups the day before, as previously published.^19^ The score can vary from 0 to 13, and the indicator was the consumption in the highest quintile of the score, corresponding to seven or more food items or groups.

Questions used to calculate health indicators are in the Supporting Information S1: Table 1.

### 2.4 Dimensions of inequality

The dimensions of inequality addressed were gender (boys and girls), area of residence (urban and rural), and socioeconomic position. The latter variable was based on the wealth index, calculated using principal component analysis (PCA), considering data about the number of household members, number of bathrooms, and existence of monthly maid/domestic employees and assets at the household, including mobile phone, computer, internet access, car, and motorcycle. The product of the PCA was later categorized into quintiles, the first representing the poorest and the fifth, the wealthiest. In addition to the variables of the dimensions of inequality, participants' age (13−15 year and 16−17 year) and geographic region (North, Northeast, Southeast, South, and Midwest) were also used to describe the sample.

### 2.5 Data analysis

First, we described the sample according to socioeconomic and demographic characteristics. Then, we described the prevalence (%) and 95% confidence intervals (95% CI) of each health indicator. To evaluate inequalities, simple and complex absolute measures were applied according to each dimension of inequality. For gender and area of residence, the inequality measure was the difference in the prevalence of the health indicators, providing the absolute gap between the two groups of each variable. For wealth, we calculated the Slope Index of Inequality (SII). The SII considers the whole distribution of the dimension of inequality, estimating the absolute difference between extreme predicted values throughout a logistic regression.^20^, ^21^ Finally, gender‐, area of residence‐ and wealth‐specific inequalities were addressed according to subnational regions (Brazilian states), applying a double stratification. The statistical significance of both difference and SII were based on their 95% CI. The 95%CI that includes value zero (absence of inequality) was considered nonsignificant. Significant differences and SIIs were presented in bold in the Figures, and Supporting Information Materials present detailed information regarding all estimates presented in the Figures.

We performed all analyses using the Stata software, version 16.1, considering the sample weights of the survey.

## 3 RESULTS

This study comprises a sample of 124,898 Brazilian adolescents aged 13−17 years. Supporting Information S1: Table 2 shows the sample distribution according to gender, age, subnational region, wealth, and area of residence. The sample includes 50.7% girls and 49.3% boys. Most part of adolescents were from Southeast (38.8%) and North−East (28.4%) regions of the country. The majority of students are aged 13−15 years (64.7%) and residents from the urban area (92.4%). Regarding wealth, 30,2% of adolescents were classified in the poorest group.

**Figure 1:**
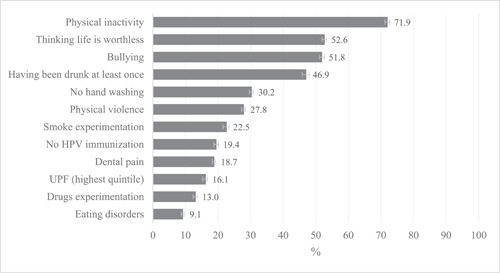
National prevalence of health indicators among Brazilian adolescents.

Figure 1 present the prevalence of each outcome in the total sample. The most prevalent outcome was physical inactivity (71.9%) followed by thinking life is worthless (52.6%), bullying (51.8%), and have been drunk at least once in life (46.9%).

**Figure 2:**
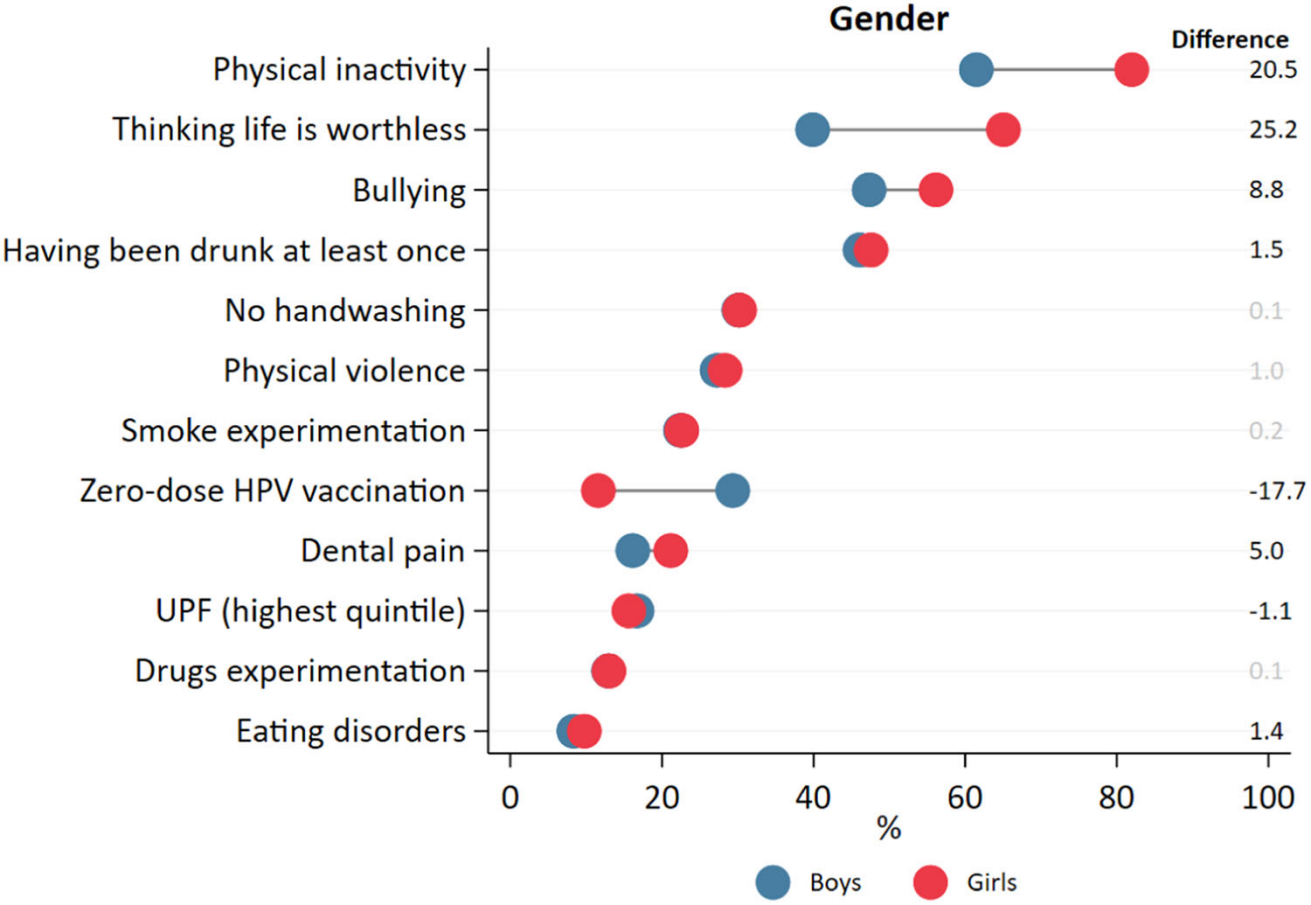
Health inequalities according to gender in Brazilian adolescents. *Note*: Statistically significant differences are presented in black. Negative values indicate higher prevalence among boys, a positive difference indicates higher prevalence among girls. Graph ordered by prevalence of outcomes.

Figure 2 and Supporting Information S1: Table 3 show inequalities according to gender for the 12 indicators sorted by prevalence. Significant gender differences are presented in bold. The most unequal indicators were thinking life is worthless, physical inactivity, and zero‐dose HPV. For the indicator thinking life is worthless, girls presented a prevalence of 25.2 p.p. higher than boys. For physical inactivity, girls also presented a prevalence of 20.5 p.p. higher than boys. On the other hand, for zero‐dose HPV, boys were the vulnerable group, presenting a prevalence of 17.7 p.p. higher than girls. For bullying, having been drunk at least once, dental pain, and eating disorders, girls presented a higher prevalence, while for UPF consumption, boys presented the highest prevalence.

**Figure 3:**
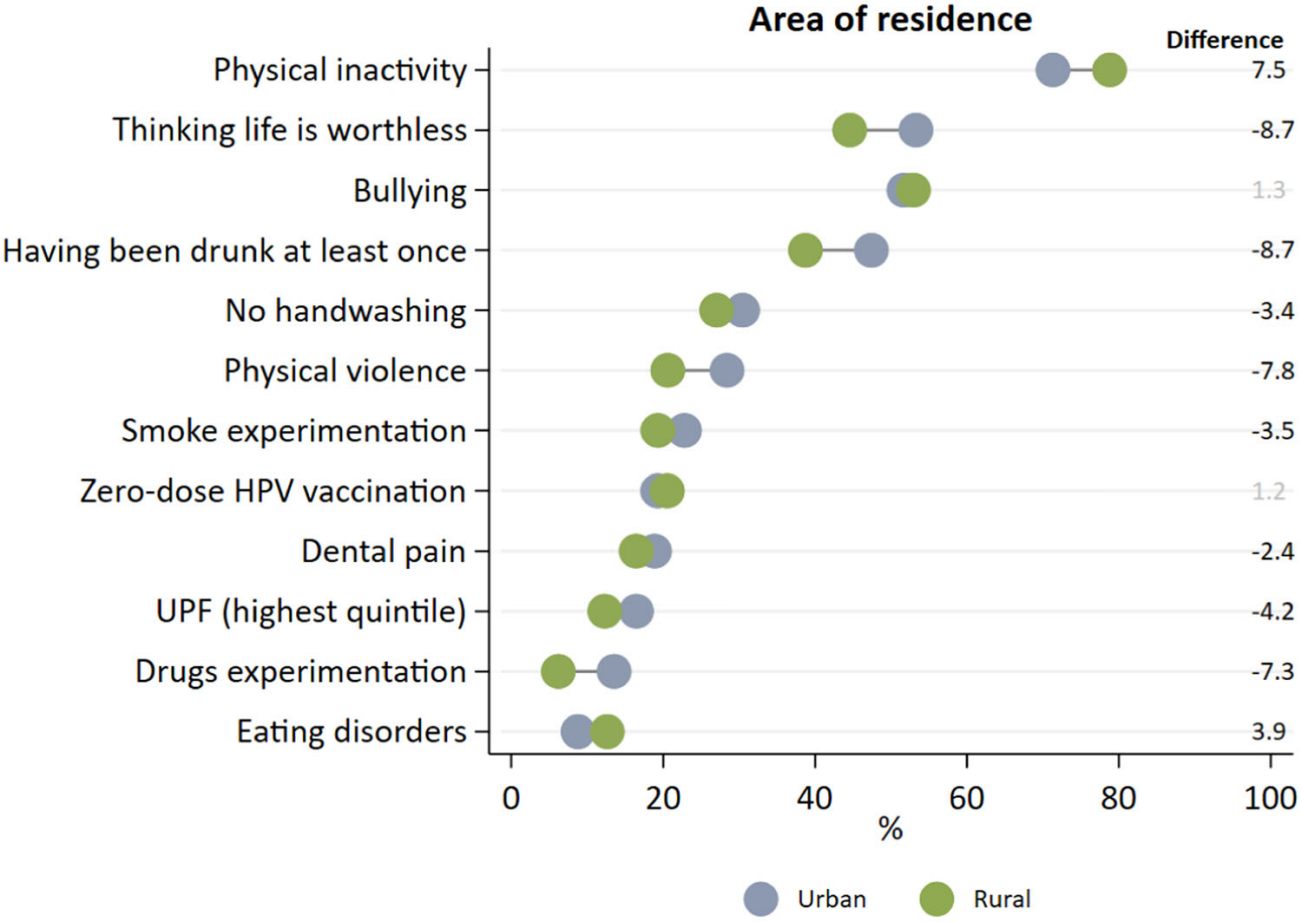
Health inequalities according to area of residence in Brazilian adolescents. *Note*: Statistically significant differences are presented in black. Negative values indicate higher prevalence among urban residents, a positive difference indicates higher prevalence among rural residents. Graph ordered by prevalence of outcomes.

Inequalities regarding area of residence are presented in Figure 3 and Supporting Information S1: Table 4. The most unequal indicators were physical inactivity, thinking life is worthless, physical violence, having been drunk at least once, and drug experimentation. For thinking is worthless, physical violence, having been drunk at least once, and drug experimentation, residents from the urban area presented 8.7, 7.8, 8.7, and 7.3 p.p. higher prevalence than residents from the urban area, respectively. In the opposite way, residents from the rural area presented a prevalence of 7.5 p.p. higher than urban residents for physical inactivity. Differences for other indicators were lower than 5 p.p. or nonsignificant.

**Figure 4:**
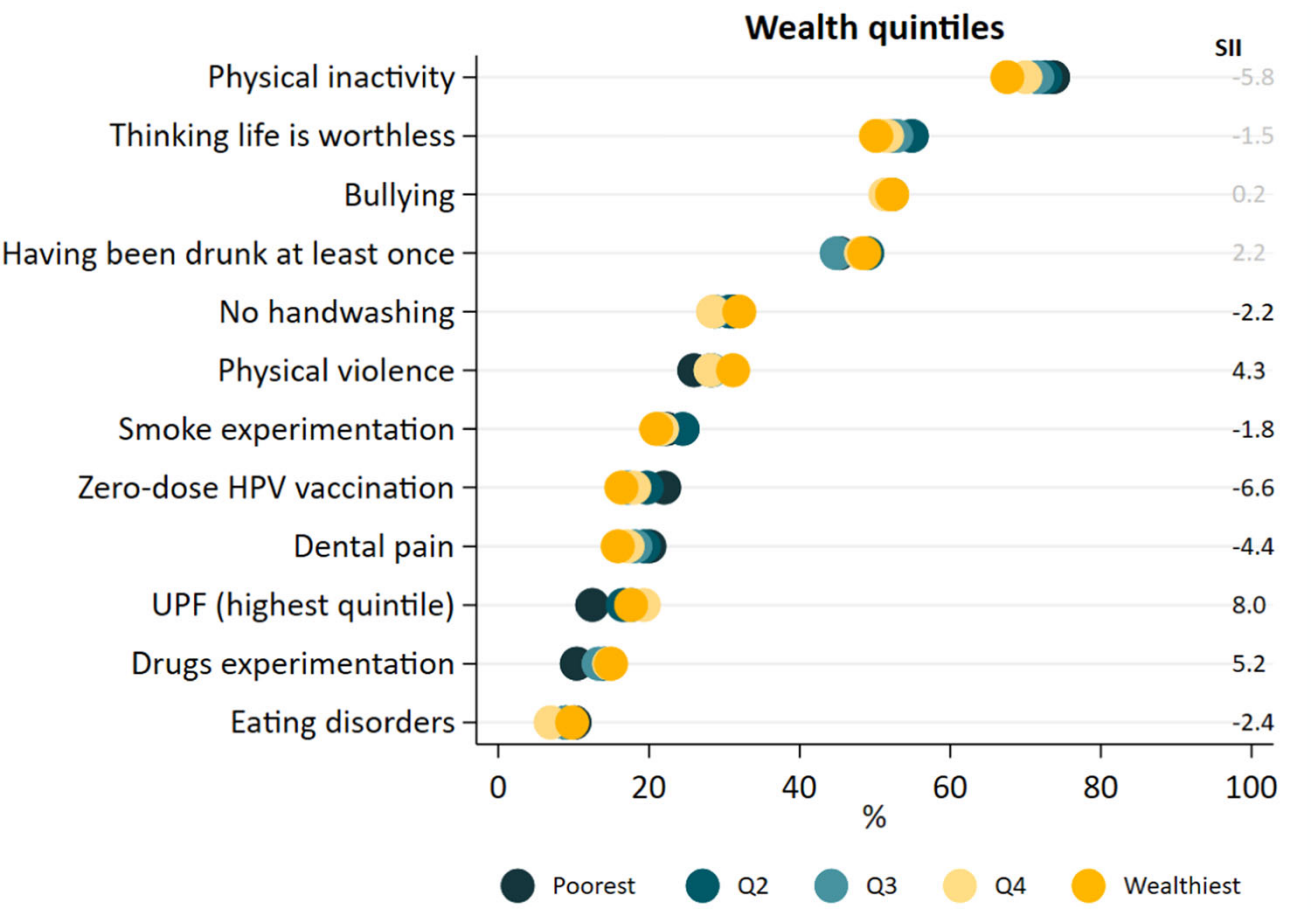
Health inequalities according to wealth quintiles in Brazilian adolescents. *Note*: Statistically significant slope index of inequality is presented in black. Negative values indicate higher prevalence among the poorest, a positive difference indicates higher prevalence among the wealthiest. Graph ordered by prevalence of outcomes.

Figure 4 and Supporting Information S1: Table 5 show wealth inequalities. The most unequal indicator was UPF consumption, with the two wealthier quintiles presenting a higher prevalence (SII = 8.0). Drug experimentation was also higher in the wealthiest (SII = 5.2), while zero‐dose HPV was more common in the poorest (SII = −6.6). Other indicators presented SII lower than 5 p.p. or were nonsignificant.

The prevalence of outcomes according to states is presented in Supporting Information S1: Table 6. The indicators with higher variability according to states were no handwashing (from 21.0% in Rio Grande do Sul to 41.3% in Ceará), smoke experimentation (from 12.9% in Bahia to 33.2% in Acre), zero‐dose HPV (from 12.9% in Espírito Santo to 29.3% in Acre), having been drunk at least once in life (from37.8% in Pará to 52.3% in Distrito Federal), and physical violence (from 21.1% in Piauí to 35.1% in Rio de Janeiro).

**Figure 5:**
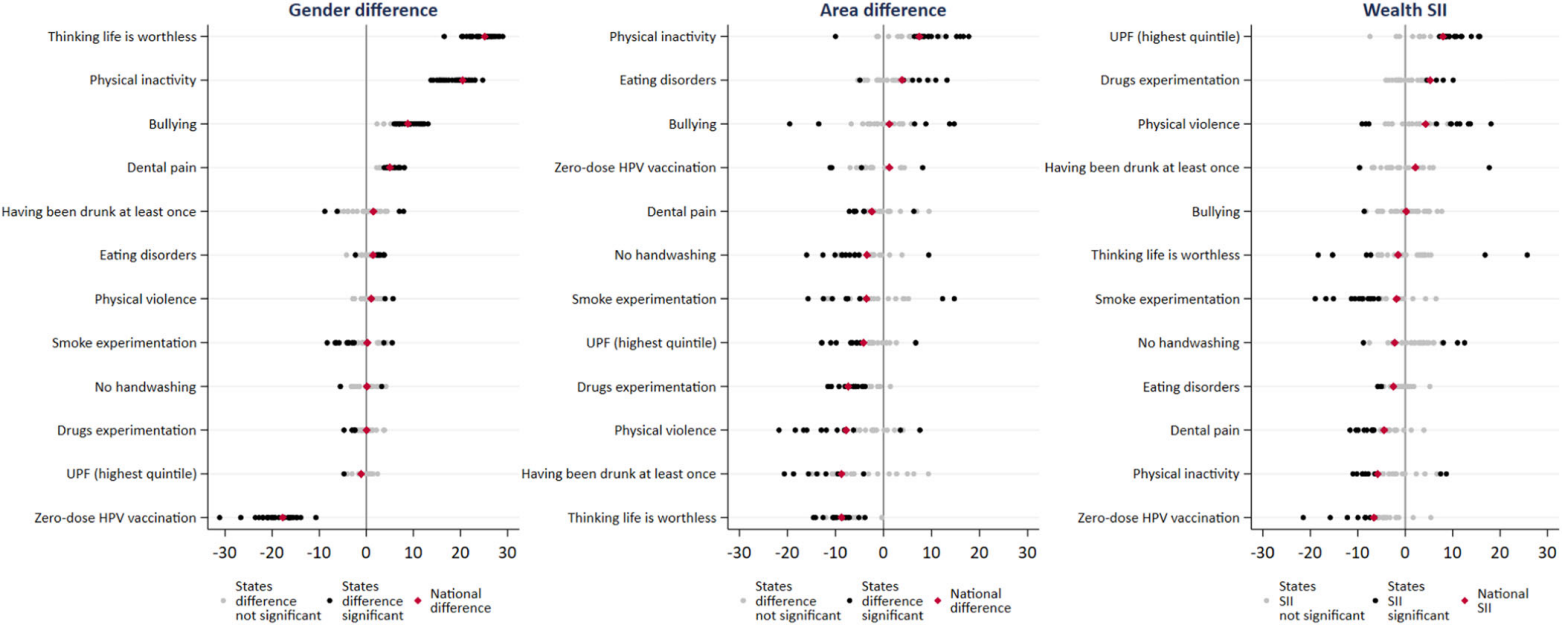
Subnational variability in inequality measures among Brazilian adolescents. *Note*: Graph ordered by national inequality measures. Significant inequality measures for states are presented in black. Gender difference: Negative values indicate a higher prevalence among boys and positive differences indicates a higher prevalence among girls. Area difference: Negative values indicate higher prevalence among urban residents, positive differences indicate a higher prevalence among rural residents. Wealth SII: Negative values indicate a higher prevalence among the poorest, positive differences indicate a higher prevalence among the wealthiest. SII, slope Index of Inequality.

Figure 5 and Supporting Information S1: Table 7 show the variability of outcomes according to gender, area of residence, and wealth inequalities in Brazilian states. Red dots represent the national inequality measure, and black dots the significant state measures.

Regarding gender inequalities, girls presented a higher prevalence of thinking life is worthless than boys in all states (following the national pattern). This difference varied widely, reaching 29.9 p.p. in Rio Grande do Sul while in Para was 16.5 p.p. Gender inequalities for physical inactivity, bullying, and dental pain followed the same pattern with higher prevalence among girls in all states with a significant difference following the direction of the national estimate. Zero‐dose HPV, higher among boys, also varied widely according to states (from −13.9 in Pará to −31.2 p.p. in Acre). For other indicators, the national gender inequality was small or nonexistent, but we found relevant results in some states.

The subnational variability according to the area of residence is also presented in Figure 5 and Supporting Information S1: Table 7. Brazilian states follow the national inequality measure for physical inactivity (higher prevalence in the rural area) except for Distrito Federal, where adolescents from the urban area presented a higher prevalence (difference = −10 p.p.). Similarly, for eating disorders (more common in the rural area), only one state (Acre) presented a prevalence of 4.9 p.p. higher in the urban area. Both national and state inequalities were higher for thinking life is worthless, having been drunk at least once, and drug experimentation in the urban area. However, these differences varied widely. Other indicators presented a small difference between rural and urban areas but high variability in the direction of these differences.

Regarding wealth inequalities, estimates by state followed the national estimate for drug experimentation and UPF consumption, with significantly higher prevalence among the richest. The inverse occurred with zero‐dose HPV and dental pain, where the national and significant states are all negative values of SII, indicating higher prevalence among the poorest. The zero‐dose HPV SII varied widely (from −6.4 in Goiás to −21.5 in Maranhão). All other indicators presented prevalence with higher variability (sometimes higher in the poorest, sometimes in the wealthiest). (Figure 5 and Supporting Information S1: Table 7).

## 4 DISCUSSION

This study presents data regarding health inequalities from 124,898 Brazilian adolescents in the most recent National Survey of School Health (2019). Gender inequalities were more marked for physical inactivity and thinking life is worthless with girls presenting a prevalence more than 20 p.p. higher than boys. In zero‐dose HPV, however, the prevalence in girls was 17.7 p.p. lower than in boys. Disparities regarding the area of residence and wealth quintiles were smaller than gender differences. In general, adolescents from the urban area presented an 8 p.p. higher prevalence of thinking life is worthless and having been drunk at least once. For wealth quintiles, the higher gap was identified for UPF, with the wealthiest presenting a prevalence 8 p.p. higher than the poorest. We also showed the relevance of context in inequality analysis stratifying results by states of the country, revealing high variability in inequality measures.

Among all dimensions of inequalities evaluated in our study, gender was the most unequal, presenting wide gaps in the prevalence of thinking life is worthless, physical inactivity, and zero‐HPV, with girls presenting a higher prevalence of unfavorable outcomes for most health indicators. Thinking life is worthless was the indicator with the widest gender gap, with a 25 p.p. difference. This indicator works as a proxy of mental health, and our results were in line with previous findings pointing out that girls usually report worse mental health than boys.^22^, ^23^ Although biological factors, such as hormonal variation and body changes, play an important role during adolescence, social aspects are the main reason behind adolescent girls' mental health. They experience a range of body‐related social pressures and even some forms of violence, which could be amplified by social media use.^24^, ^25^

Physical inactivity also showed a wide gender gap, reinforcing the worldwide reported pieces of evidence that gender is among the main determinants of physical inactivity for all age groups.^26^, ^27^, ^28^ Physical activity practice can be heavily impacted by sociocultural gender norms, especially for girls, who are culturally expected to behave in specific ways depending on the context they are inserted. Evidence from 64 global south countries showed that country‐level contextual factors, such as income level, human development index, and gender inequality index, can impact the size of the gender gap for physical activity^29^ among adolescents. Moreover, girls often have less opportunity^30^ and social support^31^ to be physically active than boys.

For most analyzed health indicators, girls showed the worst situation, having a higher prevalence of unfavorable outcomes. The only exception was that the zero HPV vaccination prevalence was higher among boys. In Brazil, HPV vaccination started with the National Immunization Program in 2014 with a target population of adolescent girls aged 9−14. In 2017, adolescent boys from 11 to 14 years were included in the target population, expanding to the same age range as girls in 2022.^32^ So, HPV vaccination was freely available, advertised, and encouraged by the Unified Health System only among girls for a long period. Despite these efforts, vaccination coverage for HPV is still low in Brazil, especially among adolescent boys, due to barriers such as unawareness regarding the existence of the vaccine, misinformation about the vaccine side effects, and low intention to vaccinate.^33^, ^34^, ^35^

Regarding urban/rural areas disaggregation, adolescents living in urban areas thought life was worthless in a higher proportion when compared with those living in rural areas. It is known that mental illness usually presents a higher prevalence in urban compared to rural areas.^36^ A recent systematic review including more than 7000 Australian youth aged between 10 and 24 reported that depression and anxiety were more prevalent in urban than rural areas. However, the difference was not statistically significant.^37^ Community belongingness may lead to better mental health status in rural adolescents.^37^ Therefore, monitoring this behavior, specially in urban adolescents, seems relevant to avoid possible future mental health disorders.

Our results showed that adolescents from the urban area reported having been drunk at least once in their life more frequently in comparison with those living in the rural area. At the same time, data from Australia and Spain showed that adolescents living in the rural area were at a higher risk.^38^, ^39^ In Brazil, alcohol consumption is possibly related to social activities that generally are less frequent in the rural area.^40^ However, the prevalence of this behavior was high for both areas, with a need for public strategies to reduce alcohol consumption in this age group. Regarding physical inactivity, a higher prevalence of insufficiently active adolescents was found in the rural area, in agreement with previous data from Portugal.^41^ This situation contrasts with previous knowledge suggesting that the rapid urbanization in Latin America and the perception of a lack of safety in the cities could lead to a lower prevalence of physical activity in urban areas.^42^ It is important to highlight that the domain in which physical activity happens is a determinant of the practice. Previous studies have shown that while rural areas are marked by work‐related physical activity, the urban area presents different opportunities for leisure‐time physical activity,^43^ an extremely relevant domain in adolescence.

We have shown that UPF consumption was higher among the wealthiest. Unlike the Global North context, where ultra‐processed products are considerably cheaper than unprocessed or minimally processed foods, prices in Brazil are still expensive and, consequently, accessible to those with a higher purchasing power.^44^ However, since the beginning of the century, UPF has been receiving successive price reductions, and according to statistical projections, they will become cheaper than healthy foods by 2026.^44^ In fact, there is evidence of a reduction in socioeconomic inequalities in UPF consumption over 10 years (2008−2017) in Brazil, with an increase in the consumption of these products among the poorest.^45^

Our study also showed that the wealthiest adolescents presented the highest prevalence of drug experimentation. The literature, on the other hand, shows that drug use is usually higher among adolescents from lower socioeconomic positions.^46^, ^47^, ^48^ In agreement with our results, lower HPV vaccination prevalence has been observed among socioeconomically disadvantaged girls.^49^ This association could be explained by lower knowledge about HPV infection and the importance of the vaccine for its prevention in this population. Discussions about HPV and vaccination could be a strategy to improve adolescents' knowledge and increase vaccination.

Our analysis of inequality stratified by Brazilian states showed high variability in the prevalence of outcomes and direction or magnitude of the measures. These results highlight that national estimates sometimes hide important findings.^50^ This variability is even more relevant when considering the large size of the country, as well as its vast population and high cultural and social diversity. Presenting the prevalence of outcomes by subnational units is also important once geographically defined regions tend to be similar in several aspects, such as availability of services, infrastructure, perceived and built environment, climate, facilities, and culture.^50^ Sometimes, only one dimension of inequality is insufficient to identify groups in higher vulnerability. In our study, we shed light not only on the variability in the frequency of outcomes but also on the double‐stratification of states and gender, wealth, and area of residence. Following this strategy, it is possible to see that, in some places, the vulnerable group differs from the most vulnerable in the country. Additionally, it allows identifying states with much higher inequality than the national measure, which could be considered in public health planning.

This study has some limitations that should be considered when interpreting the findings. Our main limitation is that health‐related outcomes were assessed using single questions or modules of questions based on self‐reported information, which could result in under/overestimating some health risk indicators, depending on whether these behaviors are socially acceptable or undesirable.^51^ Despite this limitation, we assessed data from a representative sample of Brazilian students attending public or private schools in rural or urban areas. In Brazil, the school rate from 6 to 14 years in 2019 was 99.7%, and from 15 to 17 years, 89.2%.^52^ The research findings contributed to identifying which health indicators have the largest gaps and enabled a better understanding of health inequalities according to socioeconomic, place of residence, and gender among adolescents in a middle‐income country. In addition, explorating intersectionalities with subnational regions evidenced the relevance of disaggregate data in countries such as Brazil, with great cultural diversity.

## 5 CONCLUSIONS

This study identified some health indicators with large inequalities among adolescents. We highlight the need for attention to disparities between subgroups of the adolescent population, especially for gender inequalities that were the most marked for this age group. While girls seem more vulnerable regarding lifestyle, boys should receive attention regarding healthcare (e.g., vaccination). Urban‐rural and wealth inequalities were also identified but in a lower magnitude than gender inequalities. Additionally, we highlight the subnational disparities in a country such as Brazil and suggest that interventions in the most vulnerable groups account for its cultural diversity.

## AUTHOR CONTRIBUTIONS

**Andrea Wendt**: Conceptualization; data curation; formal analysis; investigation; methodology; project administration; supervision; writing—original draft; writing—review and editing. **Adriana K. F. Machado**: Data curation; investigation; methodology; supervision; writing—original draft; writing—review and editing. **Caroline S. Costa**: Conceptualization; investigation; methodology; writing—original draft; writing—review and editing. **Daniela Rachadel**: Investigation; visualization; writing—original draft; writing—review and editing. **Inacio Crochemore‐Silva**: Investigation; supervision; visualization; writing—original draft; writing—review and editing. **Javier Brazo‐Sayavera**: Investigation; visualization; writing—original draft; writing—review and editing. **Paula K. Hembecker**: Investigation; visualization; writing—original draft; writing—review and editing. **Luiza I. C. Ricardo**: Conceptualization; investigation; methodology; supervision; visualization; writing—original draft; writing—review and editing.

## CONFLICT OF INTEREST STATEMENT

The authors declare no conflict of interest.

## TRANSPARENCY STATEMENT

The lead author Luiza I. C. Ricardo affirms that this manuscript is an honest, accurate, and transparent account of the study being reported; that no important aspects of the study have been omitted; and that any discrepancies from the study as planned (and, if relevant, registered) have been explained.

## Supporting information

Supporting information.

## Data Availability

Data from National Survey of School Health (Pesquisa Nacional de Saúde do Escolar—PeNSE) are publicly available on https://www.ibge.gov.br/estatisticas/sociais/educacao/9134-pesquisa-nacional-de-saude-do-escolar.html?=&t=microdados. National Survey of School Health (Pesquisa Nacional de Saúde do Escolar—PeNSE)'s data are available on Brazilian Institute of Statistics website (https://www.ibge.gov.br/estatisticas/sociais/populacao/9134-pesquisa-nacional-de-saude-do-escolar.html?edicao=31442&t=microdados).
