# Supplementary material for: Health inequalities in Brazilian adolescents: Measuring and mapping gaps in a cross‐sectional school‐based survey
Source: Health Sci Rep. 2023 Dec 15;6(12):e1761. doi: 10.1002/hsr2.1761 (PMC10723783; doi:10.1002/hsr2.1761)
Supplement: Supplementary file 1 — Supporting information. [file HSR2-6-e1761-s001.docx]

**Supplementary materials**

**Supplementary Table 1. Questions used to calculate the indicators.**

| **Indicator** | **Questions** |
| --- | --- |
| Physical inactivity | In the last 7 days, how many days have you walked or biked to school? How long did it take? |
|  | In the last 7 days, how many days have you walked or biked home from school? How long did it take? |
|  | In the last 7 days, how many days did you have physical education classes at school? |
|  | How much time a day have you practised physical activity or sports in the physical education classes at school? Do not consider time spent in theoretical activities. |
|  | In the last 7 days, not considering physical education classes, how many days have you practised any physical activity? How long were these activities? |
| Thinking life is worthless | In the last 30 days, how often have you felt that life is not worth living? |
| Bullying | In the last 30 days, how many times have been bullied to the level of feeling bothered, hurt, annoyed, offended, or humiliated? |
|  | In the last 30 days, how many times have your school colleagues refused talking to you, left you alone with no reason, or made other colleagues ignore you? |
|  | In the last 30 days, how many times has any of your school colleagues punched, slapped, kicked, or physically hurt you in any other way? |
| Have been drunk at least once in life | In your life up to now, how many times have you drunk to the level of feeling really drunk? |
| No hand washing | How often do you wash your hands after using the toilet? |
| Physical violence | In the last 12 months, how many times have you been physically maltreated by your mother, father, or caregiver? |
|  | In the last 12 months, how many times have you been physically maltreated by any other person apart from your mother, father, or caregiver? |
| Smoke experimentation | Have you ever smoked cigarettes in your life? Even one or two drags? |
| Zero-dose HPV immunization | Have you been vaccinated against HPV? |
| Dental pain | In the last 6 months, have you experienced dental pain not caused by braces? |
| Consumption of ultra-processed foods | Yesterday, did you drink any soft drinks? |
|  | Yesterday, did you drink any fruit juice in box or can? |
|  | Yesterday, did you drink any powdered drink mix? |
|  | Yesterday, did you drink any chocolate drink? |
|  | Yesterday, did you drink any flavored yogurt? |
|  | Yesterday, did you eat any packaged salty snacks or crackers? |
|  | Yesterday, did you eat any sweet cookies, sandwich cookies or packaged cakes? |
|  | Yesterday, did you eat any chocolate, ice cream, jelly, flan or any other industrialized desserts? |
|  | Yesterday, did you eat any sausage, mortadella or ham? |
|  | Yesterday, did you eat any loaf, hot dog or hamburger bread? |
|  | Yesterday, did you any eat margarine?  Yesterday, did you eat any mayonnaise, ketchup or other industrialized sauces? |
|  | Yesterday, did you eat any instant noodles, instant powdered soup, frozen lasagna or any other ready-to-eat frozen dish? |
| Drugs experimentation | Have you ever smoked weed or skunk, used cocaine, crack, ecstasy, oxy, or MD, sniffed glue, inhaled *lança-perfume* (similar to popper), or used any other drug? |
| Eating disorders | In the last 30 days, have you vomited or taken laxatives to lose weight or avoid gaining weight? |
|  | In the last 30 days, have you taken any medicine, custom medication, or any other product to lose weight without prescription? |

**Supplementary Table 2.** Sample characterization

| **Variables** | **n** | **% (95% CI)** |
| --- | --- | --- |
| **Sex*** |  |  |
| Male | 61,462 | 49.3 (48.5; 50.1) |
| Female | 63,148 | 50.7 (49.9; 51.5) |
| **Region** |  |  |
| North | 28,264 | 10.8 (10.0; 11.8) |
| North-East | 42,955 | 28.4 (27.2; 29.6) |
| Southeast | 22,600 | 38.8 (37.2; 40.4) |
| South | 13,439 | 13.7 (12.9; 14.6) |
| Midwest | 17,640 | 8.3 (7.8; 8.8) |
| **Age** |  |  |
| 13-15 | 82,389 | 64.7 (62.3; 66.9) |
| 16-17 | 42,509 | 35.3 (33.1; 37.6) |
| **Wealth quintiles**** |  |  |
| Poorest | 26,077 | 30.2 (29.1; 31.3) |
| Q2 | 23,782 | 24.2 (23.6; 24.9) |
| Q3 | 24,906 | 21.8 (21.1; 22.6) |
| Q4 | 25,214 | 15.6 (14.9; 16.2) |
| Wealthiest | 24,597 | 8.2 (7.8; 8.7) |
| **Area of residence** |  |  |
| Urban | 118,642 | 92.4 (91.0; 93.6) |
| Rural | 6,256 | 7.6 (6.4; 9.0) |

*Sex Variable presents missing information. N= 124,610 adolescents

**Wealth quintiles presents missing information. N= 121,576 adolescents

**Supplementary Table 3. Prevalence of indicators according to gender.**

|  | **Estimate** | **95%CI** | | **Gender** |
| --- | --- | --- | --- | --- |
| **Indicator** |  | **Lower limit** | **Upper limit** |  |
| UPF (highest quintile) | 16.7 | 16.0 | 17.5 | Boys |
| UPF (highest quintile) | 15.6 | 14.8 | 16.5 | Girls |
| Physical inactivity | 61.5 | 60.5 | 62.5 | Boys |
| Physical inactivity | 82.0 | 81.2 | 82.7 | Girls |
| Smoke experimentation | 22.4 | 21.4 | 23.4 | Boys |
| Smoke experimentation | 22.6 | 21.4 | 23.9 | Girls |
| Have been drunk at least once in life | 46.1 | 44.8 | 47.4 | Boys |
| Have been drunk at least once in life | 47.6 | 46.3 | 48.9 | Girls |
| Drugs experimentation | 12.9 | 12.2 | 13.8 | Boys |
| Drugs experimentation | 13.0 | 12.2 | 13.9 | Girls |
| No HPV immunization | 29.3 | 28.2 | 30.4 | Boys |
| No HPV immunization | 11.6 | 10.9 | 12.3 | Girls |
| Bullying | 47.3 | 46.4 | 48.3 | Boys |
| Bullying | 56.1 | 55.1 | 57.1 | Girls |
| Thinking life is worthless | 39.9 | 39.0 | 40.7 | Boys |
| Thinking life is worthless | 65.1 | 64.2 | 65.9 | Girls |
| Physical violence | 27.3 | 26.5 | 28.1 | Boys |
| Physical violence | 28.3 | 27.5 | 29.2 | Girls |
| No handwashing | 30.1 | 29.3 | 31.0 | Boys |
| No handwashing | 30.3 | 29.5 | 31.0 | Girls |
| Dental pain | 16.1 | 15.4 | 16.9 | Boys |
| Dental pain | 21.1 | 20.6 | 21.8 | Girls |
| Eating disorders | 8.4 | 7.8 | 9.0 | Boys |
| Eating disorders | 9.8 | 9.2 | 10.3 | Girls |

**Supplementary Table 4. Prevalence of indicators according to area of residence.**

|  | **Estimate** | **95%CI** | | **Area** |
| --- | --- | --- | --- | --- |
| **Indicator** |  | **Lower limit** | **Upper limit** |  |
| UPF (highest quintile) | 16.5 | 15.7 | 17.2 | Urban |
| UPF (highest quintile) | 12.3 | 10.8 | 14.0 | Rural |
| Physical inactivity | 71.3 | 70.5 | 72.1 | Urban |
| Physical inactivity | 78.8 | 76.7 | 80.8 | Rural |
| Smoke experimentation | 22.8 | 21.8 | 23.8 | Urban |
| Smoke experimentation | 19.3 | 16.7 | 22.1 | Rural |
| Have been drunk at least once in life | 47.4 | 46.3 | 48.6 | Urban |
| Have been drunk at least once in life | 38.7 | 35.5 | 42.0 | Rural |
| Drugs experimentation | 13.5 | 12.8 | 14.3 | Urban |
| Drugs experimentation | 6.2 | 5.2 | 7.4 | Rural |
| No HPV immunization | 19.3 | 18.6 | 20.0 | Urban |
| No HPV immunization | 20.5 | 17.6 | 23.8 | Rural |
| Bullying | 51.7 | 50.9 | 52.5 | Urban |
| Bullying | 53.0 | 50.3 | 55.6 | Rural |
| Thinking life is worthless | 53.3 | 52.6 | 54.0 | Urban |
| Thinking life is worthless | 44.6 | 41.7 | 47.5 | Rural |
| Physical violence | 28.4 | 27.8 | 29.0 | Urban |
| Physical violence | 20.6 | 18.8 | 22.5 | Rural |
| No handwashing | 30.5 | 29.8 | 31.1 | Urban |
| No handwashing | 27.0 | 25.2 | 29.0 | Rural |
| Dental pain | 18.9 | 18.4 | 19.4 | Urban |
| Dental pain | 16.4 | 14.9 | 18.0 | Rural |
| Eating disorders | 8.8 | 8.3 | 9.3 | Urban |
| Eating disorders | 12.6 | 10.5 | 15.1 | Rural |

**Supplementary Table 5. Prevalence of indicators according to wealth quintiles.**

|  | **Estimate** | **95%CI** | | **Wealth quintiles** |
| --- | --- | --- | --- | --- |
| **Indicator** |  | **Lower limit** | **Upper limit** |  |
| UPF (highest quintile) | 12.5 | 11.7 | 13.3 | 1rst (poorest) |
| UPF (highest quintile) | 16.6 | 15.3 | 17.9 | 2nd |
| UPF (highest quintile) | 17.7 | 16.5 | 19.0 | 3rd |
| UPF (highest quintile) | 19.3 | 18.1 | 20.6 | 4th |
| UPF (highest quintile) | 17.6 | 16.3 | 19.1 | 5th (wealthiest) |
| Physical inactivity | 73.7 | 72.4 | 74.9 | 1rst (poorest) |
| Physical inactivity | 72.6 | 71.3 | 73.9 | 2nd |
| Physical inactivity | 71.5 | 70.2 | 72.9 | 3rd |
| Physical inactivity | 70.0 | 68.4 | 71.5 | 4th |
| Physical inactivity | 67.6 | 65.8 | 69.3 | 5th (wealthiest) |
| Smoke experimentation | 22.4 | 21.2 | 23.6 | 1rst (poorest) |
| Smoke experimentation | 24.5 | 23.1 | 25.9 | 2nd |
| Smoke experimentation | 21.7 | 20.3 | 23.3 | 3rd |
| Smoke experimentation | 21.7 | 19.9 | 23.6 | 4th |
| Smoke experimentation | 21.0 | 19.3 | 22.7 | 5th (wealthiest) |
| Have been drunk at least once in life | 45.3 | 43.8 | 46.9 | 1rst (poorest) |
| Have been drunk at least once in life | 49.0 | 47.2 | 50.8 | 2nd |
| Have been drunk at least once in life | 44.9 | 42.7 | 47.2 | 3rd |
| Have been drunk at least once in life | 48.2 | 45.9 | 50.5 | 4th |
| Have been drunk at least once in life | 48.6 | 46.7 | 50.4 | 5th (wealthiest) |
| Drugs experimentation | 10.4 | 9.6 | 11.3 | 1rst (poorest) |
| Drugs experimentation | 14.1 | 13.0 | 15.3 | 2nd |
| Drugs experimentation | 13.3 | 12.1 | 14.6 | 3rd |
| Drugs experimentation | 14.7 | 13.3 | 16.3 | 4th |
| Drugs experimentation | 15.0 | 13.6 | 16.4 | 5th (wealthiest) |
| No HPV immunization | 22.0 | 20.9 | 23.2 | 1rst (poorest) |
| No HPV immunization | 19.7 | 18.6 | 20.9 | 2nd |
| No HPV immunization | 17.2 | 16.1 | 18.4 | 3rd |
| No HPV immunization | 18.0 | 16.4 | 19.8 | 4th |
| No HPV immunization | 16.4 | 15.0 | 17.8 | 5th (wealthiest) |
| Bullying | 51.8 | 50.6 | 52.9 | 1rst (poorest) |
| Bullying | 51.6 | 50.3 | 52.9 | 2nd |
| Bullying | 52.2 | 51.0 | 53.5 | 3rd |
| Bullying | 51.4 | 49.6 | 53.2 | 4th |
| Bullying | 52.3 | 50.5 | 54.1 | 5th (wealthiest) |
| Thinking life is worthless | 52.0 | 50.8 | 53.2 | 1rst (poorest) |
| Thinking life is worthless | 54.9 | 53.4 | 56.4 | 2nd |
| Thinking life is worthless | 52.8 | 51.6 | 54.1 | 3rd |
| Thinking life is worthless | 51.6 | 50.2 | 52.9 | 4th |
| Thinking life is worthless | 50.2 | 48.3 | 52.0 | 5th (wealthiest) |
| Physical violence | 26.0 | 24.9 | 27.1 | 1rst (poorest) |
| Physical violence | 28.2 | 27.1 | 29.3 | 2nd |
| Physical violence | 28.4 | 27.2 | 29.6 | 3rd |
| Physical violence | 28.2 | 26.8 | 29.6 | 4th |
| Physical violence | 31.2 | 29.6 | 32.8 | 5th (wealthiest) |
| No handwashing | 31.2 | 30.1 | 32.3 | 1rst (poorest) |
| No handwashing | 30.7 | 29.6 | 31.9 | 2nd |
| No handwashing | 28.8 | 27.7 | 29.9 | 3rd |
| No handwashing | 28.5 | 27.0 | 30.1 | 4th |
| No handwashing | 32.0 | 30.4 | 33.6 | 5th (wealthiest) |
| Dental pain | 20.1 | 19.2 | 20.9 | 1rst (poorest) |
| Dental pain | 19.3 | 18.4 | 20.3 | 2nd |
| Dental pain | 18.2 | 17.2 | 19.2 | 3rd |
| Dental pain | 17.1 | 15.8 | 18.6 | 4th |
| Dental pain | 15.9 | 14.6 | 17.2 | 5th (wealthiest) |
| Eating disorders | 10.1 | 9.3 | 11.0 | 1rst (poorest) |
| Eating disorders | 9.0 | 8.3 | 9.7 | 2nd |
| Eating disorders | 8.9 | 8.1 | 9.8 | 3rd |
| Eating disorders | 6.9 | 6.2 | 7.8 | 4th |
| Eating disorders | 9.8 | 8.9 | 10.8 | 5th (wealthiest) |

**Supplementary Table 6. Prevalence of indicators according to Brazilian states.**

| **Brazilian state** | **Indicator** | **Prevalence (%)** | **95%CI** | |
| --- | --- | --- | --- | --- |
|  |  |  | **Lower limit** | **Upper limit** |
| Rondônia | UPF (highest quintile) | 13,3 | 11,9 | 14,8 |
| Rondônia | Physical inactivity | 70,8 | 67,7 | 73,7 |
| Rondônia | Smoke experimentation | 24,8 | 21,4 | 28,7 |
| Rondônia | Have been drunk at least once in life | 49,6 | 45,4 | 53,7 |
| Rondônia | Drugs experimentation | 11,5 | 8,3 | 15,6 |
| Rondônia | No HPV immunization | 20,0 | 17,9 | 22,2 |
| Rondônia | Bullying | 50,9 | 48,0 | 53,7 |
| Rondônia | Thinking life is worthless | 54,4 | 51,4 | 57,4 |
| Rondônia | Physical violence | 24,4 | 22,4 | 26,5 |
| Rondônia | No handwashing | 26,3 | 25,0 | 27,6 |
| Rondônia | Dental pain | 21,8 | 20,0 | 23,7 |
| Rondônia | Eating disorders | 8.8 | 7.3 | 10.4 |
| Acre | UPF (highest quintile) | 17,2 | 15,3 | 19,3 |
| Acre | Physical inactivity | 70,3 | 67,9 | 72,6 |
| Acre | Smoke experimentation | 33,2 | 30,0 | 36,6 |
| Acre | Have been drunk at least once in life | 49,2 | 45,4 | 53,1 |
| Acre | Drugs experimentation | 12,5 | 10,9 | 14,3 |
| Acre | No HPV immunization | 29,3 | 27,1 | 31,6 |
| Acre | Bullying | 51,6 | 48,8 | 54,3 |
| Acre | Thinking life is worthless | 55,0 | 52,6 | 57,4 |
| Acre | Physical violence | 25,4 | 23,7 | 27,3 |
| Acre | No handwashing | 34,4 | 31,7 | 37,3 |
| Acre | Dental pain | 20,6 | 19,0 | 22,4 |
| Acre | Eating disorders | 9.7 | 8.6 | 10.9 |
| Amazonas | UPF (highest quintile) | 15,0 | 13,5 | 16,7 |
| Amazonas | Physical inactivity | 70,9 | 68,0 | 73,7 |
| Amazonas | Smoke experimentation | 24,4 | 21,9 | 27,0 |
| Amazonas | Have been drunk at least once in life | 45,8 | 41,5 | 50,1 |
| Amazonas | Drugs experimentation | 12,3 | 10,5 | 14,2 |
| Amazonas | No HPV immunization | 16,2 | 14,2 | 18,4 |
| Amazonas | Bullying | 51,9 | 48,5 | 55,3 |
| Amazonas | Thinking life is worthless | 53,3 | 49,4 | 57,1 |
| Amazonas | Physical violence | 29,0 | 27,0 | 31,0 |
| Amazonas | No handwashing | 35,4 | 33,1 | 37,7 |
| Amazonas | Dental pain | 19,6 | 17,1 | 22,4 |
| Amazonas | Eating disorders | 11.9 | 10.3 | 13.7 |
| Roraima | UPF (highest quintile) | 16,1 | 14,2 | 18,2 |
| Roraima | Physical inactivity | 72,8 | 69,7 | 75,8 |
| Roraima | Smoke experimentation | 27,1 | 24,6 | 29,8 |
| Roraima | Have been drunk at least once in life | 45,9 | 43,0 | 48,8 |
| Roraima | Drugs experimentation | 11,0 | 9,4 | 12,9 |
| Roraima | No HPV immunization | 18,9 | 16,9 | 21,1 |
| Roraima | Bullying | 48,9 | 46,1 | 51,8 |
| Roraima | Thinking life is worthless | 53,1 | 51,1 | 55,0 |
| Roraima | Physical violence | 26,9 | 24,6 | 29,3 |
| Roraima | No handwashing | 32,8 | 30,4 | 35,3 |
| Roraima | Dental pain | 21,2 | 19,6 | 22,9 |
| Roraima | Eating disorders | 12.3 | 10.5 | 14.3 |
| Para | UPF (highest quintile) | 13,0 | 11,4 | 14,8 |
| Para | Physical inactivity | 74,5 | 71,7 | 77,1 |
| Para | Smoke experimentation | 20,3 | 17,1 | 23,8 |
| Para | Have been drunk at least once in life | 37,8 | 34,5 | 41,2 |
| Para | Drugs experimentation | 7,0 | 5,5 | 8,8 |
| Para | No HPV immunization | 23,5 | 19,5 | 28,0 |
| Para | Bullying | 55,8 | 52,2 | 59,3 |
| Para | Thinking life is worthless | 53,1 | 49,9 | 56,3 |
| Para | Physical violence | 24,2 | 21,6 | 26,9 |
| Para | No handwashing | 28,5 | 26,3 | 30,8 |
| Para | Dental pain | 18,7 | 16,5 | 21,2 |
| Para | Eating disorders | 12.5 | 9.8 | 15.8 |
| Amapá | UPF (highest quintile) | 16,8 | 15,4 | 18,3 |
| Amapá | Physical inactivity | 72,1 | 70,0 | 74,1 |
| Amapá | Smoke experimentation | 25,9 | 23,2 | 28,8 |
| Amapá | Have been drunk at least once in life | 42,8 | 39,2 | 46,5 |
| Amapá | Drugs experimentation | 11,7 | 9,8 | 13,9 |
| Amapá | No HPV immunization | 26,0 | 23,6 | 28,5 |
| Amapá | Bullying | 54,8 | 52,7 | 57,0 |
| Amapá | Thinking life is worthless | 56,5 | 54,3 | 58,6 |
| Amapá | Physical violence | 27,4 | 25,7 | 29,2 |
| Amapá | No handwashing | 31,1 | 29,1 | 33,2 |
| Amapá | Dental pain | 19,7 | 18,1 | 21,3 |
| Amapá | Eating disorders | 11.9 | 10.7 | 13.2 |
| Tocantins | UPF (highest quintile) | 12,1 | 10,4 | 13,9 |
| Tocantins | Physical inactivity | 67,4 | 64,3 | 70,3 |
| Tocantins | Smoke experimentation | 22,8 | 19,4 | 26,7 |
| Tocantins | Have been drunk at least once in life | 46,4 | 42,9 | 49,9 |
| Tocantins | Drugs experimentation | 8,7 | 7,2 | 10,6 |
| Tocantins | No HPV immunization | 20,7 | 18,6 | 23,0 |
| Tocantins | Bullying | 54,4 | 51,7 | 57,1 |
| Tocantins | Thinking life is worthless | 51,7 | 49,1 | 54,3 |
| Tocantins | Physical violence | 21,5 | 19,0 | 24,2 |
| Tocantins | No handwashing | 30,1 | 27,4 | 32,9 |
| Tocantins | Dental pain | 21,3 | 19,3 | 23,4 |
| Tocantins | Eating disorders | 10.0 | 8.2 | 12.2 |
| Maranhão | UPF (highest quintile) | 12,9 | 10,8 | 15,3 |
| Maranhão | Physical inactivity | 75,9 | 73,6 | 78,2 |
| Maranhão | Smoke experimentation | 18,7 | 15,6 | 22,1 |
| Maranhão | Have been drunk at least once in life | 41,3 | 37,0 | 45,8 |
| Maranhão | Drugs experimentation | 8,2 | 6,7 | 9,8 |
| Maranhão | No HPV immunization | 26,5 | 23,3 | 30,0 |
| Maranhão | Bullying | 50,5 | 46,8 | 54,2 |
| Maranhão | Thinking life is worthless | 46,6 | 43,5 | 49,6 |
| Maranhão | Physical violence | 23,2 | 20,2 | 26,4 |
| Maranhão | No handwashing | 28,1 | 26,0 | 30,4 |
| Maranhão | Dental pain | 19,2 | 17,0 | 21,5 |
| Maranhão | Eating disorders | 11.1 | 9.5 | 12.8 |
| Piauí | UPF (highest quintile) | 12,2 | 10,4 | 14,2 |
| Piauí | Physical inactivity | 77,4 | 74,9 | 79,7 |
| Piauí | Smoke experimentation | 17,7 | 15,2 | 20,5 |
| Piauí | Have been drunk at least once in life | 42,0 | 38,0 | 46,1 |
| Piauí | Drugs experimentation | 7,4 | 5,7 | 9,5 |
| Piauí | No HPV immunization | 20,7 | 18,8 | 22,8 |
| Piauí | Bullying | 51,7 | 49,2 | 54,3 |
| Piauí | Thinking life is worthless | 48,2 | 45,9 | 50,5 |
| Piauí | Physical violence | 21,1 | 19,0 | 23,3 |
| Piauí | No handwashing | 35,2 | 32,4 | 38,0 |
| Piauí | Dental pain | 19,6 | 17,8 | 21,4 |
| Piauí | Eating disorders | 6.7 | 5.6 | 8.0 |
| Ceará | UPF (highest quintile) | 14,6 | 11,9 | 17,9 |
| Ceará | Physical inactivity | 76,2 | 72,8 | 79,2 |
| Ceará | Smoke experimentation | 17,6 | 14,9 | 20,6 |
| Ceará | Have been drunk at least once in life | 43,6 | 40,4 | 46,9 |
| Ceará | Drugs experimentation | 10,0 | 8,2 | 12,0 |
| Ceará | No HPV immunization | 20,5 | 17,4 | 24,0 |
| Ceará | Bullying | 52,7 | 49,6 | 55,8 |
| Ceará | Thinking life is worthless | 52,8 | 50,1 | 55,4 |
| Ceará | Physical violence | 26,1 | 23,9 | 28,5 |
| Ceará | No handwashing | 41,3 | 39,2 | 43,5 |
| Ceará | Dental pain | 18,2 | 16,3 | 20,4 |
| Ceará | Eating disorders | 8.5 | 6.8 | 10.5 |
| Rio Grande do Norte | UPF (highest quintile) | 15,4 | 14,0 | 17,0 |
| Rio Grande do Norte | Physical inactivity | 73,9 | 72,1 | 75,7 |
| Rio Grande do Norte | Smoke experimentation | 15,0 | 13,2 | 17,1 |
| Rio Grande do Norte | Have been drunk at least once in life | 39,7 | 36,6 | 42,8 |
| Rio Grande do Norte | Drugs experimentation | 9,3 | 7,8 | 11,1 |
| Rio Grande do Norte | No HPV immunization | 26,8 | 24,0 | 29,9 |
| Rio Grande do Norte | Bullying | 50,7 | 48,1 | 53,3 |
| Rio Grande do Norte | Thinking life is worthless | 50,5 | 48,3 | 52,6 |
| Rio Grande do Norte | Physical violence | 26,1 | 24,1 | 28,2 |
| Rio Grande do Norte | No handwashing | 38,9 | 36,5 | 41,4 |
| Rio Grande do Norte | Dental pain | 15,1 | 13,4 | 16,9 |
| Rio Grande do Norte | Eating disorders | 7.8 | 6.5 | 9.2 |
| Paraíba | UPF (highest quintile) | 17,3 | 15,6 | 19,1 |
| Paraíba | Physical inactivity | 76,8 | 74,6 | 78,8 |
| Paraíba | Smoke experimentation | 18,3 | 16,4 | 20,4 |
| Paraíba | Have been drunk at least once in life | 42,9 | 40,1 | 45,7 |
| Paraíba | Drugs experimentation | 8,9 | 7,6 | 10,5 |
| Paraíba | No HPV immunization | 22,8 | 20,2 | 25,7 |
| Paraíba | Bullying | 53,4 | 51,1 | 55,7 |
| Paraíba | Thinking life is worthless | 49,1 | 46,7 | 51,6 |
| Paraíba | Physical violence | 24,7 | 22,9 | 26,6 |
| Paraíba | No handwashing | 37,4 | 35,3 | 39,6 |
| Paraíba | Dental pain | 17,2 | 15,7 | 18,9 |
| Paraíba | Eating disorders | 8.2 | 7.0 | 9.7 |
| Pernambuco | UPF (highest quintile) | 16,8 | 15,2 | 18,7 |
| Pernambuco | Physical inactivity | 76,2 | 73,4 | 78,8 |
| Pernambuco | Smoke experimentation | 18,9 | 16,6 | 21,3 |
| Pernambuco | Have been drunk at least once in life | 42,9 | 39,5 | 46,4 |
| Pernambuco | Drugs experimentation | 9,0 | 7,2 | 11,2 |
| Pernambuco | No HPV immunization | 22,6 | 19,8 | 25,5 |
| Pernambuco | Bullying | 52,9 | 49,7 | 56,0 |
| Pernambuco | Thinking life is worthless | 52,3 | 49,3 | 55,3 |
| Pernambuco | Physical violence | 26,0 | 23,5 | 28,6 |
| Pernambuco | No handwashing | 34,8 | 32,7 | 36,9 |
| Pernambuco | Dental pain | 17,9 | 16,4 | 19,5 |
| Pernambuco | Eating disorders | 10.0 | 8.4 | 11.9 |
| Alagoas | UPF (highest quintile) | 15,6 | 13,9 | 17,6 |
| Alagoas | Physical inactivity | 77,9 | 75,3 | 80,3 |
| Alagoas | Smoke experimentation | 18,9 | 15,7 | 22,6 |
| Alagoas | Have been drunk at least once in life | 42,0 | 38,7 | 45,3 |
| Alagoas | Drugs experimentation | 6,6 | 5,2 | 8,2 |
| Alagoas | No HPV immunization | 23,3 | 20,7 | 26,1 |
| Alagoas | Bullying | 55,3 | 52,2 | 58,4 |
| Alagoas | Thinking life is worthless | 51,5 | 48,8 | 54,1 |
| Alagoas | Physical violence | 23,5 | 20,9 | 26,2 |
| Alagoas | No handwashing | 33,9 | 31,1 | 36,9 |
| Alagoas | Dental pain | 19,7 | 18,1 | 21,4 |
| Alagoas | Eating disorders | 8.0 | 6.3 | 10.1 |
| Sergipe | UPF (highest quintile) | 12,4 | 11,3 | 13,6 |
| Sergipe | Physical inactivity | 75,8 | 73,0 | 78,4 |
| Sergipe | Smoke experimentation | 13,6 | 12,0 | 15,4 |
| Sergipe | Have been drunk at least once in life | 42,6 | 39,6 | 45,5 |
| Sergipe | Drugs experimentation | 7,2 | 5,9 | 8,8 |
| Sergipe | No HPV immunization | 21,4 | 19,1 | 23,9 |
| Sergipe | Bullying | 49,4 | 46,3 | 52,6 |
| Sergipe | Thinking life is worthless | 53,1 | 50,8 | 55,4 |
| Sergipe | Physical violence | 25,1 | 22,8 | 27,5 |
| Sergipe | No handwashing | 31,9 | 29,6 | 34,2 |
| Sergipe | Dental pain | 16,1 | 14,3 | 18,0 |
| Sergipe | Eating disorders | 7.8 | 6.7 | 9.2 |
| Bahia | UPF (highest quintile) | 13,3 | 11,2 | 15,7 |
| Bahia | Physical inactivity | 74,6 | 71,9 | 77,1 |
| Bahia | Smoke experimentation | 12,9 | 10,7 | 15,4 |
| Bahia | Have been drunk at least once in life | 42,5 | 38,8 | 46,2 |
| Bahia | Drugs experimentation | 5,5 | 4,3 | 7,0 |
| Bahia | No HPV immunization | 16,9 | 14,7 | 19,4 |
| Bahia | Bullying | 45,5 | 42,7 | 48,3 |
| Bahia | Thinking life is worthless | 51,1 | 48,8 | 53,4 |
| Bahia | Physical violence | 24,8 | 22,3 | 27,5 |
| Bahia | No handwashing | 28,6 | 26,5 | 30,9 |
| Bahia | Dental pain | 17,6 | 15,9 | 19,5 |
| Bahia | Eating disorders | 8.6 | 7.3 | 10.1 |
| Minas Gerais | UPF (highest quintile) | 15,5 | 13,5 | 17,8 |
| Minas Gerais | Physical inactivity | 69,3 | 66,8 | 71,6 |
| Minas Gerais | Smoke experimentation | 22,2 | 19,7 | 24,9 |
| Minas Gerais | Have been drunk at least once in life | 46,5 | 42,8 | 50,3 |
| Minas Gerais | Drugs experimentation | 12,5 | 10,7 | 14,7 |
| Minas Gerais | No HPV immunization | 14,7 | 12,6 | 16,9 |
| Minas Gerais | Bullying | 48,1 | 44,9 | 51,3 |
| Minas Gerais | Thinking life is worthless | 49,8 | 46,7 | 52,9 |
| Minas Gerais | Physical violence | 28,2 | 25,6 | 30,8 |
| Minas Gerais | No handwashing | 29,3 | 27,1 | 31,6 |
| Minas Gerais | Dental pain | 17,8 | 16,3 | 19,4 |
| Minas Gerais | Eating disorders | 8.6 | 7.1 | 10.3 |
| Espirito Santo | UPF (highest quintile) | 13,6 | 12,1 | 15,2 |
| Espirito Santo | Physical inactivity | 72,0 | 70,2 | 73,7 |
| Espirito Santo | Smoke experimentation | 23,1 | 20,4 | 26,0 |
| Espirito Santo | Have been drunk at least once in life | 48,1 | 44,2 | 52,0 |
| Espirito Santo | Drugs experimentation | 16,0 | 13,5 | 18,8 |
| Espirito Santo | No HPV immunization | 12,9 | 11,1 | 15,1 |
| Espirito Santo | Bullying | 50,6 | 48,1 | 53,2 |
| Espirito Santo | Thinking life is worthless | 55,8 | 53,8 | 57,7 |
| Espirito Santo | Physical violence | 25,9 | 24,0 | 27,8 |
| Espirito Santo | No handwashing | 27,7 | 26,1 | 29,4 |
| Espirito Santo | Dental pain | 16,3 | 14,9 | 17,8 |
| Espirito Santo | Eating disorders | 7.9 | 6.8 | 9.2 |
| Rio de Janeiro | UPF (highest quintile) | 16,7 | 15,4 | 18,0 |
| Rio de Janeiro | Physical inactivity | 74,2 | 72,3 | 76,0 |
| Rio de Janeiro | Smoke experimentation | 23,1 | 20,9 | 25,4 |
| Rio de Janeiro | Have been drunk at least once in life | 49,5 | 46,3 | 52,8 |
| Rio de Janeiro | Drugs experimentation | 14,9 | 13,1 | 17,0 |
| Rio de Janeiro | No HPV immunization | 24,2 | 22,1 | 26,5 |
| Rio de Janeiro | Bullying | 51,1 | 48,9 | 53,4 |
| Rio de Janeiro | Thinking life is worthless | 55,3 | 53,3 | 57,3 |
| Rio de Janeiro | Physical violence | 35,1 | 33,1 | 37,2 |
| Rio de Janeiro | No handwashing | 35,7 | 33,8 | 37,7 |
| Rio de Janeiro | Dental pain | 16,9 | 15,7 | 18,2 |
| Rio de Janeiro | Eating disorders | 9.5 | 8.3 | 10.9 |
| São Paulo | UPF (highest quintile) | 19,2 | 16,7 | 22,1 |
| São Paulo | Physical inactivity | 69,8 | 67,2 | 72,3 |
| São Paulo | Smoke experimentation | 25,0 | 21,4 | 29,0 |
| São Paulo | Have been drunk at least once in life | 49,7 | 45,9 | 53,5 |
| São Paulo | Drugs experimentation | 18,3 | 15,4 | 21,6 |
| São Paulo | No HPV immunization | 17,8 | 15,9 | 19,9 |
| São Paulo | Bullying | 55,0 | 52,8 | 57,2 |
| São Paulo | Thinking life is worthless | 53,7 | 51,3 | 56,0 |
| São Paulo | Physical violence | 31,9 | 30,2 | 33,6 |
| São Paulo | No handwashing | 29,7 | 27,8 | 31,7 |
| São Paulo | Dental pain | 20,5 | 19,0 | 22,1 |
| São Paulo | Eating disorders | 8.9 | 7.4 | 10.6 |
| Paraná | UPF (highest quintile) | 18,6 | 16,6 | 20,9 |
| Paraná | Physical inactivity | 66,1 | 63,4 | 68,6 |
| Paraná | Smoke experimentation | 31,6 | 28,4 | 34,9 |
| Paraná | Have been drunk at least once in life | 51,0 | 47,9 | 54,0 |
| Paraná | Drugs experimentation | 18,9 | 16,4 | 21,7 |
| Paraná | No HPV immunization | 14,4 | 12,1 | 17,1 |
| Paraná | Bullying | 52,3 | 48,9 | 55,6 |
| Paraná | Thinking life is worthless | 54,6 | 52,2 | 57,0 |
| Paraná | Physical violence | 28,0 | 26,0 | 30,1 |
| Paraná | No handwashing | 24,8 | 22,5 | 27,3 |
| Paraná | Dental pain | 18,9 | 17,1 | 20,8 |
| Paraná | Eating disorders | 9.6 | 7.4 | 12.3 |
| Santa Catarina | UPF (highest quintile) | 16,2 | 14,5 | 18,1 |
| Santa Catarina | Physical inactivity | 66,4 | 63,3 | 69,3 |
| Santa Catarina | Smoke experimentation | 27,9 | 24,4 | 31,7 |
| Santa Catarina | Have been drunk at least once in life | 50,0 | 45,9 | 54,1 |
| Santa Catarina | Drugs experimentation | 16,4 | 14,0 | 19,0 |
| Santa Catarina | No HPV immunization | 16,4 | 14,3 | 18,8 |
| Santa Catarina | Bullying | 51,3 | 48,3 | 54,2 |
| Santa Catarina | Thinking life is worthless | 54,4 | 51,8 | 57,0 |
| Santa Catarina | Physical violence | 26,3 | 23,0 | 29,9 |
| Santa Catarina | No handwashing | 25,3 | 22,8 | 27,9 |
| Santa Catarina | Dental pain | 17,0 | 15,5 | 18,7 |
| Santa Catarina | Eating disorders | 8.5 | 7.2 | 10.0 |
| Rio Grande do Sul | UPF (highest quintile) | 17,9 | 15,9 | 20,1 |
| Rio Grande do Sul | Physical inactivity | 69,9 | 65,4 | 73,9 |
| Rio Grande do Sul | Smoke experimentation | 25,6 | 22,5 | 29,0 |
| Rio Grande do Sul | Have been drunk at least once in life | 50,1 | 46,2 | 54,1 |
| Rio Grande do Sul | Drugs experimentation | 14,5 | 12,3 | 17,0 |
| Rio Grande do Sul | No HPV immunization | 19,0 | 16,1 | 22,4 |
| Rio Grande do Sul | Bullying | 49,7 | 46,3 | 53,2 |
| Rio Grande do Sul | Thinking life is worthless | 52,7 | 49,3 | 56,0 |
| Rio Grande do Sul | Physical violence | 25,4 | 22,7 | 28,2 |
| Rio Grande do Sul | No handwashing | 21,0 | 19,0 | 23,2 |
| Rio Grande do Sul | Dental pain | 17,9 | 15,9 | 20,2 |
| Rio Grande do Sul | Eating disorders | 6.2 | 5.2 | 7.3 |
| Mato Grosso do Sul | UPF (highest quintile) | 15,0 | 12,6 | 17,8 |
| Mato Grosso do Sul | Physical inactivity | 69,1 | 65,8 | 72,2 |
| Mato Grosso do Sul | Smoke experimentation | 31,4 | 27,7 | 35,4 |
| Mato Grosso do Sul | Have been drunk at least once in life | 51,0 | 46,9 | 55,0 |
| Mato Grosso do Sul | Drugs experimentation | 14,4 | 12,4 | 16,7 |
| Mato Grosso do Sul | No HPV immunization | 22,2 | 18,9 | 26,0 |
| Mato Grosso do Sul | Bullying | 52,4 | 49,7 | 55,0 |
| Mato Grosso do Sul | Thinking life is worthless | 55,9 | 53,2 | 58,5 |
| Mato Grosso do Sul | Physical violence | 29,6 | 26,6 | 32,7 |
| Mato Grosso do Sul | No handwashing | 24,9 | 22,9 | 26,9 |
| Mato Grosso do Sul | Dental pain | 19,1 | 16,9 | 21,5 |
| Mato Grosso do Sul | Eating disorders | 10.0 | 8.0 | 12.5 |
| Mato Grosso | UPF (highest quintile) | 15,3 | 13,3 | 17,5 |
| Mato Grosso | Physical inactivity | 72,3 | 68,8 | 75,5 |
| Mato Grosso | Smoke experimentation | 25,7 | 22,5 | 29,2 |
| Mato Grosso | Have been drunk at least once in life | 46,7 | 41,6 | 51,8 |
| Mato Grosso | Drugs experimentation | 9,7 | 7,8 | 12,0 |
| Mato Grosso | No HPV immunization | 20,7 | 17,7 | 24,0 |
| Mato Grosso | Bullying | 50,0 | 47,8 | 52,2 |
| Mato Grosso | Thinking life is worthless | 51,8 | 49,5 | 54,0 |
| Mato Grosso | Physical violence | 22,7 | 20,5 | 25,1 |
| Mato Grosso | No handwashing | 25,4 | 22,9 | 28,2 |
| Mato Grosso | Dental pain | 21,5 | 19,6 | 23,6 |
| Mato Grosso | Eating disorders | 9.1 | 7.6 | 70.8 |
| Goiás | UPF (highest quintile) | 15,4 | 13,5 | 17,6 |
| Goiás | Physical inactivity | 70,1 | 67,5 | 72,7 |
| Goiás | Smoke experimentation | 24,6 | 22,5 | 26,8 |
| Goiás | Have been drunk at least once in life | 47,2 | 44,7 | 49,7 |
| Goiás | Drugs experimentation | 14,0 | 12,3 | 15,8 |
| Goiás | No HPV immunization | 20,4 | 17,8 | 23,3 |
| Goiás | Bullying | 54,0 | 51,9 | 56,1 |
| Goiás | Thinking life is worthless | 53,5 | 51,1 | 55,8 |
| Goiás | Physical violence | 27,1 | 25,1 | 29,3 |
| Goiás | No handwashing | 26,1 | 24,1 | 28,2 |
| Goiás | Dental pain | 19,6 | 17,5 | 21,8 |
| Goiás | Eating disorders | 9.0 | 8.1 | 10.1 |
| Distrito Federal | UPF (highest quintile) | 16,6 | 14,8 | 18,5 |
| Distrito Federal | Physical inactivity | 71,6 | 69,0 | 74,0 |
| Distrito Federal | Smoke experimentation | 27,3 | 23,6 | 31,3 |
| Distrito Federal | Have been drunk at least once in life | 52,3 | 49,4 | 55,3 |
| Distrito Federal | Drugs experimentation | 21,0 | 17,6 | 24,8 |
| Distrito Federal | No HPV immunization | 18,7 | 16,9 | 20,7 |
| Distrito Federal | Bullying | 50,4 | 46,9 | 53,9 |
| Distrito Federal | Thinking life is worthless | 55,4 | 52,5 | 58,4 |
| Distrito Federal | Physical violence | 29,1 | 26,1 | 32,3 |
| Distrito Federal | No handwashing | 31,2 | 29,0 | 33,5 |
| Distrito Federal | Dental pain | 17,0 | 15,0 | 19,2 |
| Distrito Federal | Eating disorders | 7.7 | 6.3 | 9.2 |

**Supplementary Table 7. Gender, area of residence and wealth inequalities according to Brazilian states.**

| **Brazilian state** | **Indicator** | **Gender difference** | **95%CI** | | **Area  difference** | **95% CI** | | **Wealth  slope index  of inequality** | **95%CI** | |
| --- | --- | --- | --- | --- | --- | --- | --- | --- | --- | --- |
|  |  |  | **Lower limit** | **Upper limit** |  | **Lower limit** | **Upper limit** |  | **Lower limit** | **Upper limit** |
| Rondônia | UPF (highest quintile) | 0.7 | -2.4 | 3.7 | -2.5 | -5.8 | 0.8 | 8.7 | 4.0 | 13.4 |
| Rondônia | Physical inactivity | 19.3 | 14.5 | 24.2 | 9.3 | 1.6 | 16.9 | -1.9 | -8.5 | 4.8 |
| Rondônia | Smoke experimentation | -3.9 | -8.0 | 0.3 | -7.5 | -12.9 | -2.1 | 1.6 | -5.0 | 8.2 |
| Rondônia | Have been drunk at least once in life | 0.4 | -4.2 | 4.9 | -6.1 | -12.9 | 0.6 | 2.9 | -6.2 | 12.0 |
| Rondônia | Drugs experimentation | -1.0 | -3.0 | 1.1 | -8.0 | -12.4 | -3.7 | 8.0 | 3.2 | 12.8 |
| Rondônia | No HPV immunization | -16.3 | -20.6 | -12.0 | -2.7 | -9.1 | 3.6 | -4.2 | -11.1 | 2.7 |
| Rondônia | Bullying | 12.3 | 7.7 | 17.0 | 3.3 | -3.7 | 10.4 | -0.1 | -7.6 | 7.4 |
| Rondônia | Thinking life is worthless | 28.0 | 22.5 | 33.5 | -8.4 | -15.0 | -1.8 | 2.5 | -5.0 | 10.0 |
| Rondônia | Physical violence | 0.0 | -3.9 | 4.0 | -5.0 | -11.9 | 1.9 | 0.7 | -5.4 | 6.9 |
| Rondônia | No handwashing | 3.3 | 0.5 | 6.1 | -0.8 | -4.3 | 2.6 | 3.9 | -2.5 | 10.3 |
| Rondônia | Dental pain | 7.2 | 4.5 | 9.8 | -0.9 | -6.1 | 4.3 | -4.1 | -10.3 | 2.1 |
| Rondônia | Eating disorders | 0.8 | -1.4 | 3.0 | 4.2 | -0.7 | 9.0 | 0.4 | -3.7 | 4.6 |
| Acre | UPF (highest quintile) | 1.4 | -0.9 | 3.8 | -9.8 | -15.8 | -3.9 | 15.7 | 10.1 | 21.4 |
| Acre | Physical inactivity | 13.7 | 9.9 | 17.5 | 5.7 | -0.3 | 11.8 | 8.7 | 1.3 | 16.0 |
| Acre | Smoke experimentation | -3.7 | -8.2 | 0.8 | 12.3 | 0.4 | 24.2 | -19.0 | -26.4 | -11.5 |
| Acre | Have been drunk at least once in life | 3.1 | -3.2 | 9.3 | -3.1 | -12.1 | 5.9 | 0.1 | -10.0 | 10.2 |
| Acre | Drugs experimentation | -2.2 | -5.4 | 0.9 | 1.5 | -4.1 | 7.0 | -1.9 | -7.2 | 3.4 |
| Acre | No HPV immunization | -31.2 | -36.2 | -26.1 | -5.0 | -11.9 | 1.8 | -5.7 | -13.8 | 2.5 |
| Acre | Bullying | 7.0 | 2.4 | 11.6 | -19.5 | -29.7 | -9.3 | 7.7 | 0.0 | 15.5 |
| Acre | Thinking life is worthless | 25.3 | 21.7 | 28.9 | -10.4 | -20.4 | -0.5 | 0.2 | -7.6 | 8.0 |
| Acre | Physical violence | 0.5 | -3.3 | 4.3 | -13.0 | -17.1 | -8.9 | 6.2 | -0.4 | 12.8 |
| Acre | No handwashing | 0.3 | -3.6 | 4.2 | -2.9 | -11.6 | 5.9 | 8.0 | 0.8 | 15.3 |
| Acre | Dental pain | 6.8 | 3.5 | 10.1 | -4.1 | -9.7 | 1.5 | -5.3 | -11.7 | 1.1 |
| Acre | Eating disorders | 3.1 | 0.7 | 5.4 | -4.9 | -8.4 | -1.4 | -0.4 | -4.8 | 4.1 |
| Amazonas | UPF (highest quintile) | 0.1 | -4.4 | 4.7 | -1.2 | -5.6 | 3.3 | 8.0 | 1.5 | 14.5 |
| Amazonas | Physical inactivity | 15.6 | 11.8 | 19.4 | 1.1 | -5.9 | 8.1 | 6.7 | -1.8 | 15.1 |
| Amazonas | Smoke experimentation | -3.1 | -7.6 | 1.4 | 5.2 | -0.8 | 11.3 | -8.9 | -17.1 | -0.8 |
| Amazonas | Have been drunk at least once in life | -5.7 | -11.9 | 0.4 | -6.7 | -17.4 | 4.0 | 3.8 | -8.8 | 16.5 |
| Amazonas | Drugs experimentation | -1.1 | -4.6 | 2.3 | -2.9 | -7.2 | 1.3 | 3.5 | -2.2 | 9.2 |
| Amazonas | No HPV immunization | -14.7 | -18.9 | -10.5 | 1.1 | -4.2 | 6.4 | -5.8 | -13.6 | 1.9 |
| Amazonas | Bullying | 2.3 | -2.4 | 7.0 | 2.2 | -6.7 | 11.2 | -5.3 | -14.7 | 4.0 |
| Amazonas | Thinking life is worthless | 22.2 | 17.4 | 26.9 | -14.6 | -22.9 | -6.3 | 25.7 | 17.0 | 34.4 |
| Amazonas | Physical violence | 1.6 | -4.9 | 8.0 | -4.9 | -10.4 | 0.6 | 18.1 | 9.7 | 26.5 |
| Amazonas | No handwashing | -3.0 | -7.9 | 1.9 | -8.7 | -13.5 | -3.8 | 7.7 | -1.2 | 16.6 |
| Amazonas | Dental pain | 3.0 | -0.2 | 6.3 | -7.1 | -12.5 | -1.7 | 4.0 | -3.2 | 11.1 |
| Amazonas | Eating disorders |  |  |  |  |  |  |  |  |  |
| Roraima | UPF (highest quintile) | 0.3 | -3.0 | 3.7 | -5.5 | -9.4 | -1.5 | 14.0 | 8.7 | 19.2 |
| Roraima | Physical inactivity | 15.0 | 11.5 | 18.5 | 5.6 | -0.4 | 11.7 | 4.2 | -2.1 | 10.4 |
| Roraima | Smoke experimentation | -8.3 | -12.2 | -4.4 | -1.2 | -7.2 | 4.9 | -16.7 | -23.0 | -10.5 |
| Roraima | Have been drunk at least once in life | -1.9 | -7.2 | 3.3 | 1.2 | -5.4 | 7.7 | -9.6 | -19.0 | -0.3 |
| Roraima | Drugs experimentation | -4.7 | -7.4 | -2.1 | -3.0 | -6.5 | 0.5 | -4.1 | -8.8 | 0.7 |
| Roraima | No HPV immunization | -15.9 | -19.6 | -12.2 | 3.7 | -0.4 | 7.9 | -15.8 | -22.4 | -9.3 |
| Roraima | Bullying | 7.0 | 2.2 | 11.8 | 0.0 | -6.5 | 6.5 | 4.5 | -2.6 | 11.6 |
| Roraima | Thinking life is worthless | 22.2 | 17.7 | 26.6 | -5.1 | -9.7 | -0.6 | 3.7 | -3.4 | 10.8 |
| Roraima | Physical violence | -0.4 | -4.6 | 3.8 | -2.3 | -6.9 | 2.2 | 0.8 | -5.5 | 7.1 |
| Roraima | No handwashing | -1.4 | -5.5 | 2.6 | -5.1 | -10.2 | -0.1 | -0.7 | -7.3 | 5.8 |
| Roraima | Dental pain | 2.7 | -1.5 | 7.0 | -4.0 | -7.9 | -0.1 | -6.9 | -12.8 | -0.9 |
| Roraima | Eating disorders | -1.1 | -3.5 | 1.3 | 7.4 | 2.8 | 12.0 | -5.8 | -10.6 | -1.0 |
| Para | UPF (highest quintile) | 0.2 | -3.8 | 4.2 | -2.1 | -7.1 | 2.8 | 15.6 | 9.1 | 22.1 |
| Para | Physical inactivity | 18.9 | 13.5 | 24.2 | -1.4 | -8.6 | 5.8 | -3.4 | -13.2 | 6.4 |
| Para | Smoke experimentation | -4.1 | -7.2 | -0.9 | 2.6 | -7.5 | 12.7 | -8.6 | -18.0 | 0.7 |
| Para | Have been drunk at least once in life | -6.2 | -11.9 | -0.5 | -6.2 | -17.5 | 5.1 | -1.1 | -14.9 | 12.7 |
| Para | Drugs experimentation | -2.4 | -4.1 | -0.6 | -0.7 | -5.7 | 4.3 | 0.7 | -4.6 | 6.0 |
| Para | No HPV immunization | -13.9 | -20.3 | -7.5 | 3.8 | -9.7 | 17.3 | -8.6 | -20.6 | 3.5 |
| Para | Bullying | 9.4 | 4.1 | 14.7 | 5.8 | -1.8 | 13.4 | 6.8 | -4.5 | 18.1 |
| Para | Thinking life is worthless | 16.5 | 11.9 | 21.1 | -6.4 | -18.3 | 5.6 | 4.0 | -7.4 | 15.5 |
| Para | Physical violence | 1.3 | -2.3 | 5.0 | -2.0 | -8.7 | 4.7 | 9.0 | -0.1 | 18.0 |
| Para | No handwashing | 0.0 | -4.0 | 4.0 | -2.1 | -8.8 | 4.6 | -0.7 | -11.0 | 9.6 |
| Para | Dental pain | 3.8 | 0.7 | 6.9 | -6.2 | -12.1 | -0.3 | 1.3 | -7.3 | 9.8 |
| Para | Eating disorders | -4.2 | -9.8 | 1.3 | 13.2 | 1.8 | 24.7 | -5.2 | -14.2 | 3.8 |
| Amapá | UPF (highest quintile) | 0.3 | -2.6 | 3.1 | -2.5 | -7.3 | 2.4 | 7.8 | 3.1 | 12.4 |
| Amapá | Physical inactivity | 16.6 | 13.3 | 19.9 | 8.5 | 3.5 | 13.5 | 7.5 | 2.0 | 13.0 |
| Amapá | Smoke experimentation | -0.7 | -4.2 | 2.7 | -7.5 | -12.9 | -2.1 | -9.1 | -14.5 | -3.7 |
| Amapá | Have been drunk at least once in life | 2.3 | -3.5 | 8.1 | -9.5 | -17.6 | -1.4 | 1.9 | -6.1 | 10.0 |
| Amapá | Drugs experimentation | 1.4 | -1.0 | 3.8 | -7.4 | -11.0 | -3.9 | 1.4 | -2.7 | 5.5 |
| Amapá | No HPV immunization | -22.0 | -25.7 | -18.3 | -2.6 | -9.9 | 4.7 | -7.4 | -13.3 | -1.5 |
| Amapá | Bullying | 12.0 | 8.0 | 16.0 | -1.2 | -7.0 | 4.5 | 2.3 | -3.8 | 8.3 |
| Amapá | Thinking life is worthless | 25.7 | 21.5 | 30.0 | -8.6 | -14.1 | -3.1 | 3.4 | -2.6 | 9.4 |
| Amapá | Physical violence | 3.1 | 0.0 | 6.2 | -6.3 | -10.7 | -1.8 | 6.6 | 1.1 | 12.1 |
| Amapá | No handwashing | -0.2 | -3.1 | 2.7 | -10.1 | -14.4 | -5.8 | 12.5 | 7.0 | 18.1 |
| Amapá | Dental pain | 4.5 | 1.7 | 7.2 | -4.1 | -7.8 | -0.4 | -3.3 | -8.2 | 1.7 |
| Amapá | Eating disorders | 1.1 | -1.5 | 3.6 | 4.2 | 0.7 | 7.8 | -2.2 | -6.2 | 1.9 |
| Tocantins | UPF (highest quintile) | 0.4 | -2.2 | 2.9 | -11.0 | -14.0 | -8.0 | 11.7 | 6.4 | 17.1 |
| Tocantins | Physical inactivity | 17.5 | 13.6 | 21.4 | 7.0 | 0.9 | 13.2 | -0.6 | -8.6 | 7.3 |
| Tocantins | Smoke experimentation | -5.8 | -11.1 | -0.4 | -11.8 | -28.3 | 4.7 | -6.9 | -14.1 | 0.3 |
| Tocantins | Have been drunk at least once in life | -3.9 | -10.9 | 3.1 | -20.7 | -41.8 | 0.4 | -6.7 | -17.3 | 3.9 |
| Tocantins | Drugs experimentation | -2.6 | -5.9 | 0.7 | -6.1 | -9.9 | -2.4 | 1.4 | -3.4 | 6.2 |
| Tocantins | No HPV immunization | -18.6 | -22.9 | -14.2 | -3.9 | -10.9 | 3.1 | -4.6 | -12.6 | 3.4 |
| Tocantins | Bullying | 7.0 | 2.4 | 11.5 | -13.5 | -22.3 | -4.7 | 0.7 | -7.7 | 9.2 |
| Tocantins | Thinking life is worthless | 24.1 | 18.8 | 29.4 | -10.6 | -23.0 | 1.7 | -0.5 | -9.0 | 8.0 |
| Tocantins | Physical violence | 0.0 | -3.6 | 3.5 | -11.9 | -19.2 | -4.7 | -0.6 | -7.5 | 6.3 |
| Tocantins | No handwashing | 2.7 | -1.4 | 6.8 | 3.9 | -8.2 | 15.9 | 3.8 | -3.6 | 11.3 |
| Tocantins | Dental pain | 5.3 | 1.9 | 8.7 | -0.5 | -13.7 | 12.7 | -2.1 | -9.2 | 4.9 |
| Tocantins | Eating disorders | 1.7 | -1.2 | 4.7 | 1.9 | -1.1 | 4.9 | 1.8 | -3.0 | 6.6 |
| Maranhão | UPF (highest quintile) | -3.0 | -6.7 | 0.6 | -3.0 | -7.7 | 1.6 | 15.5 | 9.2 | 21.7 |
| Maranhão | Physical inactivity | 18.2 | 14.8 | 21.7 | 3.0 | -1.9 | 7.9 | -2.6 | -11.4 | 6.1 |
| Maranhão | Smoke experimentation | -6.3 | -10.7 | -2.0 | -2.0 | -8.7 | 4.7 | 6.5 | -1.3 | 14.3 |
| Maranhão | Have been drunk at least once in life | -4.8 | -11.0 | 1.4 | -7.8 | -16.5 | 1.0 | 17.7 | 4.8 | 30.7 |
| Maranhão | Drugs experimentation | -3.0 | -5.5 | -0.6 | -4.4 | -7.3 | -1.5 | 10.1 | 5.1 | 15.1 |
| Maranhão | No HPV immunization | -19.7 | -23.8 | -15.5 | 4.4 | -1.9 | 10.7 | -21.5 | -32.8 | -10.2 |
| Maranhão | Bullying | 11.7 | 5.9 | 17.5 | 1.1 | -6.6 | 8.7 | 2.7 | -7.6 | 12.9 |
| Maranhão | Thinking life is worthless | 20.6 | 15.2 | 25.9 | -10.1 | -17.9 | -2.3 | 16.9 | 7.0 | 26.7 |
| Maranhão | Physical violence | -2.5 | -6.6 | 1.6 | -3.7 | -9.4 | 1.9 | 11.5 | 3.5 | 19.5 |
| Maranhão | No handwashing | 0.5 | -3.8 | 4.8 | -3.4 | -9.3 | 2.4 | 11.0 | 2.7 | 19.4 |
| Maranhão | Dental pain | 4.7 | 0.6 | 8.8 | 1.0 | -4.3 | 6.2 | -0.3 | -8.1 | 7.4 |
| Maranhão | Eating disorders | -1.9 | -5.2 | 1.4 | 0.7 | -3.6 | 4.9 | 1.8 | -4.8 | 8.4 |
| Piauí | UPF (highest quintile) | 0.7 | -1.9 | 3.3 | -3.0 | -6.9 | 0.9 | 7.2 | 1.7 | 12.7 |
| Piauí | Physical inactivity | 15.5 | 11.4 | 19.5 | 8.3 | 0.6 | 16.1 | -1.9 | -8.8 | 5.0 |
| Piauí | Smoke experimentation | -3.7 | -7.2 | -0.2 | -3.7 | -10.2 | 2.9 | -7.4 | -14.0 | -0.8 |
| Piauí | Have been drunk at least once in life | 4.5 | -1.7 | 10.6 | -13.6 | -29.2 | 1.9 | -5.1 | -16.0 | 5.7 |
| Piauí | Drugs experimentation | -1.8 | -4.0 | 0.3 | -2.8 | -6.8 | 1.3 | 1.4 | -2.9 | 5.6 |
| Piauí | No HPV immunization | -23.6 | -27.5 | -19.6 | 1.4 | -5.6 | 8.3 | -12.2 | -20.0 | -4.4 |
| Piauí | Bullying | 11.4 | 7.0 | 15.8 | -6.7 | -17.7 | 4.2 | 5.1 | -3.2 | 13.3 |
| Piauí | Thinking life is worthless | 23.8 | 18.1 | 29.5 | -12.6 | -21.0 | -4.2 | 4.2 | -4.1 | 12.4 |
| Piauí | Physical violence | -0.9 | -4.6 | 2.8 | -9.6 | -14.7 | -4.6 | 13.3 | 7.2 | 19.5 |
| Piauí | No handwashing | 4.2 | -0.8 | 9.2 | -7.9 | -15.5 | -0.3 | 3.3 | -4.5 | 11.1 |
| Piauí | Dental pain | 5.1 | 2.0 | 8.2 | -0.2 | -5.5 | 5.2 | -4.2 | -10.9 | 2.4 |
| Piauí | Eating disorders | -2.3 | -4.2 | -0.5 | -0.2 | -4.8 | 4.4 | -0.7 | -4.8 | 3.3 |
| Ceará | UPF (highest quintile) | -1.4 | -5.3 | 2.5 | -2.1 | -6.8 | 2.6 | 8.3 | 3.0 | 13.6 |
| Ceará | Physical inactivity | 23.1 | 18.1 | 28.1 | 10.0 | 5.4 | 14.5 | -9.0 | -16.7 | -1.4 |
| Ceará | Smoke experimentation | -0.1 | -3.0 | 2.8 | -2.9 | -10.4 | 4.6 | -7.2 | -13.2 | -1.3 |
| Ceará | Have been drunk at least once in life | -2.9 | -8.1 | 2.4 | -10.3 | -20.7 | 0.2 | -2.8 | -14.8 | 9.1 |
| Ceará | Drugs experimentation | -0.3 | -3.9 | 3.2 | -6.2 | -10.0 | -2.4 | -0.9 | -5.6 | 3.9 |
| Ceará | No HPV immunization | -20.8 | -25.5 | -16.2 | -3.6 | -11.3 | 4.0 | -8.4 | -16.3 | -0.4 |
| Ceará | Bullying | 13.1 | 6.8 | 19.4 | 4.0 | -6.7 | 14.7 | -3.0 | -11.9 | 6.0 |
| Ceará | Thinking life is worthless | 20.3 | 15.5 | 25.0 | -5.8 | -12.8 | 1.2 | -5.0 | -13.9 | 4.0 |
| Ceará | Physical violence | 0.5 | -5.9 | 6.8 | -8.0 | -12.5 | -3.6 | 2.4 | -4.9 | 9.7 |
| Ceará | No handwashing | 1.4 | -4.5 | 7.2 | -6.0 | -11.4 | -0.6 | 2.1 | -6.9 | 11.0 |
| Ceará | Dental pain | 2.8 | -0.9 | 6.5 | -5.8 | -9.7 | -1.8 | -3.3 | -10.5 | 3.9 |
| Ceará | Eating disorders | -0.8 | -4.2 | 2.6 | -1.3 | -5.9 | 3.3 | -5.0 | -9.8 | -0.3 |
| Rio Grande do Norte | UPF (highest quintile) | -1.4 | -4.6 | 1.7 | -3.1 | -10.8 | 4.7 | 1.6 | -3.6 | 6.8 |
| Rio Grande do Norte | Physical inactivity | 22.0 | 19.5 | 24.5 | 6.5 | 0.7 | 12.2 | -8.4 | -14.9 | -1.8 |
| Rio Grande do Norte | Smoke experimentation | -0.4 | -4.6 | 3.8 | -7.7 | -12.8 | -2.5 | -5.3 | -11.1 | 0.5 |
| Rio Grande do Norte | Have been drunk at least once in life | -0.3 | -6.4 | 5.8 | -14.9 | -30.0 | 0.2 | 5.9 | -3.9 | 15.7 |
| Rio Grande do Norte | Drugs experimentation | 0.0 | -3.0 | 3.0 | -7.7 | -10.7 | -4.6 | 1.3 | -3.1 | 5.7 |
| Rio Grande do Norte | No HPV immunization | -22.8 | -27.1 | -18.5 | -2.2 | -14.6 | 10.1 | -1.4 | -9.0 | 6.3 |
| Rio Grande do Norte | Bullying | 7.0 | 2.2 | 11.7 | 8.8 | 2.6 | 15.0 | 4.8 | -2.7 | 12.4 |
| Rio Grande do Norte | Thinking life is worthless | 21.9 | 16.6 | 27.2 | -10.5 | -14.8 | -6.2 | -8.2 | -15.7 | -0.6 |
| Rio Grande do Norte | Physical violence | 2.9 | -0.7 | 6.4 | 4.1 | -3.4 | 11.5 | 9.7 | 3.2 | 16.2 |
| Rio Grande do Norte | No handwashing | -2.7 | -6.7 | 1.3 | -0.4 | -11.4 | 10.6 | 0.3 | -7.1 | 7.7 |
| Rio Grande do Norte | Dental pain | 4.6 | 1.6 | 7.6 | 1.2 | -7.1 | 9.6 | -4.3 | -10.0 | 1.4 |
| Rio Grande do Norte | Eating disorders | 1.6 | -0.6 | 3.8 | -0.8 | -4.4 | 2.8 | -0.3 | -4.4 | 3.7 |
| Paraíba | UPF (highest quintile) | 0.7 | -2.6 | 4.1 | -2.7 | -9.4 | 4.1 | 10.4 | 5.0 | 15.9 |
| Paraíba | Physical inactivity | 17.0 | 14.0 | 20.0 | 7.8 | 2.7 | 13.0 | -2.5 | -8.7 | 3.7 |
| Paraíba | Smoke experimentation | 0.3 | -2.6 | 3.2 | -4.3 | -11.5 | 2.9 | -9.7 | -15.9 | -3.5 |
| Paraíba | Have been drunk at least once in life | 0.4 | -4.6 | 5.4 | 2.7 | -7.1 | 12.6 | -3.5 | -12.8 | 5.8 |
| Paraíba | Drugs experimentation | 0.0 | -2.7 | 2.7 | -4.3 | -7.6 | -1.0 | -1.5 | -5.6 | 2.7 |
| Paraíba | No HPV immunization | -21.9 | -26.4 | -17.5 | -11.1 | -17.7 | -4.6 | -5.4 | -12.6 | 1.9 |
| Paraíba | Bullying | 9.3 | 5.8 | 12.8 | -2.7 | -10.3 | 4.8 | 4.0 | -3.4 | 11.4 |
| Paraíba | Thinking life is worthless | 21.3 | 17.5 | 25.1 | -0.3 | -9.0 | 8.3 | -1.1 | -8.5 | 6.3 |
| Paraíba | Physical violence | 0.9 | -2.9 | 4.6 | -8.1 | -13.1 | -3.1 | 4.7 | -1.5 | 10.8 |
| Paraíba | No handwashing | 2.2 | -1.4 | 5.9 | -9.2 | -21.7 | 3.3 | 2.8 | -4.3 | 9.9 |
| Paraíba | Dental pain | 2.2 | -1.2 | 5.6 | -2.8 | -8.9 | 3.3 | -3.7 | -9.3 | 1.9 |
| Paraíba | Eating disorders | 0.2 | -2.5 | 2.9 | 3.1 | -3.9 | 10.2 | -1.5 | -5.8 | 2.8 |
| Pernambuco | UPF (highest quintile) | -1.6 | -5.1 | 1.9 | 0.7 | -8.3 | 9.7 | 10.8 | 5.3 | 16.4 |
| Pernambuco | Physical inactivity | 15.9 | 12.2 | 19.6 | 3.6 | -3.3 | 10.6 | -7.8 | -14.7 | -0.9 |
| Pernambuco | Smoke experimentation | 2.8 | -0.6 | 6.2 | -4.9 | -8.3 | -1.5 | -0.3 | -6.5 | 5.9 |
| Pernambuco | Have been drunk at least once in life | 0.8 | -4.8 | 6.4 | -13.9 | -20.0 | -7.7 | -1.6 | -11.7 | 8.5 |
| Pernambuco | Drugs experimentation | 0.3 | -3.6 | 4.2 | -1.2 | -5.7 | 3.2 | 3.2 | -0.8 | 7.3 |
| Pernambuco | No HPV immunization | -20.9 | -27.5 | -14.2 | 1.0 | -6.2 | 8.3 | -4.8 | -12.6 | 3.1 |
| Pernambuco | Bullying | 9.5 | 4.5 | 14.4 | -0.3 | -13.1 | 12.5 | -5.8 | -13.9 | 2.2 |
| Pernambuco | Thinking life is worthless | 22.5 | 17.0 | 28.1 | -8.6 | -22.0 | 4.7 | -1.2 | -9.3 | 6.9 |
| Pernambuco | Physical violence | 1.0 | -2.6 | 4.5 | -5.7 | -12.1 | 0.6 | 9.6 | 3.1 | 16.1 |
| Pernambuco | No handwashing | -3.2 | -8.2 | 1.8 | -12.6 | -20.9 | -4.4 | 5.0 | -2.6 | 12.5 |
| Pernambuco | Dental pain | 6.1 | 3.0 | 9.2 | -3.4 | -10.1 | 3.4 | -2.2 | -8.3 | 3.9 |
| Pernambuco | Eating disorders | 0.7 | -3.7 | 5.1 | 10.9 | 3.2 | 18.6 | -0.8 | -6.1 | 4.5 |
| Alagoas | UPF (highest quintile) | 2.4 | -1.5 | 6.2 | 6.7 | 1.5 | 12.0 | 8.4 | 2.5 | 14.3 |
| Alagoas | Physical inactivity | 14.2 | 11.5 | 16.9 | 5.4 | -4.8 | 15.5 | -9.0 | -16.1 | -1.8 |
| Alagoas | Smoke experimentation | -4.3 | -9.4 | 0.7 | -7.0 | -14.3 | 0.4 | -4.0 | -11.3 | 3.3 |
| Alagoas | Have been drunk at least once in life | -8.8 | -14.5 | -3.1 | -18.7 | -28.4 | -9.1 | -1.3 | -12.8 | 10.3 |
| Alagoas | Drugs experimentation | -2.6 | -5.0 | -0.2 | -5.4 | -8.0 | -2.8 | 4.1 | 0.0 | 8.2 |
| Alagoas | No HPV immunization | -26.7 | -31.7 | -21.6 | 8.2 | 3.1 | 13.2 | -7.5 | -16.4 | 1.4 |
| Alagoas | Bullying | 3.7 | -1.1 | 8.5 | 13.7 | 6.4 | 21.1 | -0.6 | -9.6 | 8.3 |
| Alagoas | Thinking life is worthless | 22.5 | 19.5 | 25.5 | -0.3 | -10.1 | 9.4 | 4.8 | -4.1 | 13.8 |
| Alagoas | Physical violence | -0.5 | -4.2 | 3.3 | 7.6 | 0.2 | 15.0 | 3.1 | -4.1 | 10.4 |
| Alagoas | No handwashing | -1.9 | -6.3 | 2.5 | -2.0 | -8.7 | 4.8 | -7.5 | -16.0 | 0.9 |
| Alagoas | Dental pain | 8.0 | 4.5 | 11.6 | -1.8 | -8.7 | 5.0 | -3.4 | -10.4 | 3.6 |
| Alagoas | Eating disorders | -0.1 | -2.9 | 2.6 | -1.1 | -9.0 | 6.9 | 0.8 | -3.8 | 5.3 |
| Sergipe | UPF (highest quintile) | -1.1 | -3.9 | 1.8 | -4.8 | -9.3 | -0.4 | 3.1 | -1.2 | 7.4 |
| Sergipe | Physical inactivity | 19.7 | 16.7 | 22.7 | 11.3 | 4.5 | 18.1 | -6.3 | -12.3 | -0.4 |
| Sergipe | Smoke experimentation | -3.0 | -5.7 | -0.2 | -2.7 | -8.5 | 3.2 | -4.1 | -9.2 | 1.0 |
| Sergipe | Have been drunk at least once in life | -0.6 | -6.6 | 5.4 | -15.6 | -25.4 | -5.9 | -2.8 | -11.7 | 6.2 |
| Sergipe | Drugs experimentation | -1.7 | -4.3 | 0.9 | -3.8 | -7.3 | -0.3 | 4.6 | 1.2 | 8.0 |
| Sergipe | No HPV immunization | -19.9 | -23.5 | -16.3 | -2.3 | -11.4 | 6.7 | 1.7 | -4.9 | 8.3 |
| Sergipe | Bullying | 3.7 | -1.2 | 8.7 | 1.6 | -8.1 | 11.3 | 1.0 | -6.1 | 8.2 |
| Sergipe | Thinking life is worthless | 23.4 | 19.4 | 27.3 | -7.4 | -14.1 | -0.7 | 5.4 | -1.7 | 12.6 |
| Sergipe | Physical violence | -2.9 | -6.1 | 0.4 | 2.3 | -9.6 | 14.2 | 10.9 | 5.1 | 16.7 |
| Sergipe | No handwashing | -0.1 | -3.2 | 2.9 | -2.5 | -15.4 | 10.4 | 2.0 | -4.6 | 8.5 |
| Sergipe | Dental pain | 4.6 | 2.1 | 7.1 | -0.9 | -6.8 | 5.0 | -1.8 | -6.9 | 3.3 |
| Sergipe | Eating disorders | 1.3 | -1.0 | 3.5 | 2.5 | -3.9 | 9.0 | -1.0 | -4.4 | 2.5 |
| Bahia | UPF (highest quintile) | 1.1 | -1.1 | 3.2 | -6.8 | -11.0 | -2.7 | 3.9 | -2.0 | 9.8 |
| Bahia | Physical inactivity | 19.6 | 15.8 | 23.5 | 9.4 | 1.1 | 17.7 | -11.0 | -18.3 | -3.8 |
| Bahia | Smoke experimentation | -2.6 | -4.9 | -0.2 | -7.8 | -13.4 | -2.1 | 4.3 | -1.2 | 9.7 |
| Bahia | Have been drunk at least once in life | -3.9 | -11.1 | 3.3 | -4.3 | -18.4 | 9.9 | 5.9 | -4.7 | 16.5 |
| Bahia | Drugs experimentation | -0.1 | -1.8 | 1.6 | -4.0 | -6.2 | -1.8 | 6.6 | 2.9 | 10.3 |
| Bahia | No HPV immunization | -14.9 | -20.5 | -9.3 | -4.1 | -14.6 | 6.5 | -8.4 | -15.6 | -1.1 |
| Bahia | Bullying | 6.3 | 2.0 | 10.6 | -4.3 | -11.5 | 2.9 | 5.1 | -3.5 | 13.7 |
| Bahia | Thinking life is worthless | 23.7 | 18.8 | 28.6 | -14.1 | -22.7 | -5.6 | 3.0 | -5.7 | 11.6 |
| Bahia | Physical violence | 0.3 | -4.0 | 4.6 | -16.0 | -21.0 | -10.9 | 13.8 | 6.8 | 20.7 |
| Bahia | No handwashing | 0.7 | -3.2 | 4.5 | -8.5 | -13.9 | -3.1 | 4.5 | -3.1 | 12.2 |
| Bahia | Dental pain | 4.3 | 1.6 | 7.0 | -6.1 | -10.7 | -1.6 | -4.4 | -11.2 | 2.3 |
| Bahia | Eating disorders | 1.2 | -1.8 | 4.2 | -0.8 | -4.8 | 3.2 | 1.0 | -4.1 | 6.1 |
| Minas Gerais | UPF (highest quintile) | -1.4 | -4.7 | 1.8 | -12.9 | -19.8 | -5.9 | 11.9 | 6.5 | 17.4 |
| Minas Gerais | Physical inactivity | 22.4 | 18.2 | 26.6 | 11.8 | -5.6 | 29.2 | -6.6 | -14.0 | 0.8 |
| Minas Gerais | Smoke experimentation | 2.5 | -1.6 | 6.6 | -15.7 | -20.8 | -10.6 | -1.1 | -7.6 | 5.4 |
| Minas Gerais | Have been drunk at least once in life | 3.0 | -3.0 | 8.9 | -1.2 | -23.4 | 21.0 | 0.8 | -9.5 | 11.0 |
| Minas Gerais | Drugs experimentation | -0.2 | -3.4 | 2.9 | -5.8 | -10.6 | -1.0 | 3.7 | -1.4 | 8.8 |
| Minas Gerais | No HPV immunization | -17.4 | -21.1 | -13.7 | 0.2 | -10.1 | 10.6 | -10.0 | -17.5 | -2.4 |
| Minas Gerais | Bullying | 10.1 | 4.8 | 15.4 | 3.3 | -13.0 | 19.6 | -2.1 | -10.5 | 6.2 |
| Minas Gerais | Thinking life is worthless | 27.5 | 23.6 | 31.3 | -7.1 | -11.6 | -2.6 | -1.7 | -10.0 | 6.6 |
| Minas Gerais | Physical violence | 3.3 | -1.4 | 8.1 | -21.8 | -26.8 | -16.7 | 5.0 | -2.3 | 12.4 |
| Minas Gerais | No handwashing | 1.1 | -2.5 | 4.6 | 9.4 | 3.7 | 15.2 | 1.2 | -6.2 | 8.6 |
| Minas Gerais | Dental pain | 4.1 | 1.0 | 7.3 | 9.5 | -2.9 | 21.8 | -11.6 | -18.4 | -4.9 |
| Minas Gerais | Eating disorders | 2.3 | -1.1 | 5.6 | 5.3 | -3.6 | 14.2 | -1.9 | -7.2 | 3.4 |
| Espirito Santo | UPF (highest quintile) | -4.7 | -7.7 | -1.8 | -5.7 | -9.5 | -1.8 | 8.7 | 3.6 | 13.8 |
| Espirito Santo | Physical inactivity | 20.0 | 15.6 | 24.4 | 8.2 | -1.7 | 18.0 | -8.4 | -15.2 | -1.6 |
| Espirito Santo | Smoke experimentation | -0.9 | -4.9 | 3.2 | -10.7 | -16.9 | -4.4 | -9.1 | -15.7 | -2.5 |
| Espirito Santo | Have been drunk at least once in life | 1.7 | -3.1 | 6.5 | -20.6 | -30.9 | -10.4 | 3.7 | -5.7 | 13.1 |
| Espirito Santo | Drugs experimentation | -0.7 | -3.9 | 2.5 | -11.4 | -15.4 | -7.5 | -1.9 | -7.4 | 3.6 |
| Espirito Santo | No HPV immunization | -15.5 | -19.6 | -11.4 | -5.6 | -12.2 | 1.0 | -3.8 | -9.5 | 1.9 |
| Espirito Santo | Bullying | 8.2 | 3.3 | 13.1 | 0.1 | -11.1 | 11.4 | 1.6 | -6.0 | 9.3 |
| Espirito Santo | Thinking life is worthless | 25.6 | 20.9 | 30.3 | -8.3 | -17.4 | 0.8 | -7.4 | -15.0 | 0.1 |
| Espirito Santo | Physical violence | -0.1 | -5.4 | 5.2 | 0.1 | -7.7 | 8.0 | 3.0 | -3.5 | 9.5 |
| Espirito Santo | No handwashing | 0.6 | -3.9 | 5.1 | -7.1 | -13.9 | -0.2 | -2.1 | -8.9 | 4.6 |
| Espirito Santo | Dental pain | 6.9 | 4.2 | 9.6 | -6.1 | -11.5 | -0.6 | -5.0 | -10.7 | 0.8 |
| Espirito Santo | Eating disorders | -0.2 | -2.5 | 2.1 | -1.4 | -4.5 | 1.7 | -1.4 | -5.5 | 2.6 |
| Rio de Janeiro | UPF (highest quintile) | -1.1 | -4.0 | 1.7 | 1.3 | -4.7 | 7.4 | 9.4 | 4.8 | 13.9 |
| Rio de Janeiro | Physical inactivity | 20.4 | 17.6 | 23.1 | -1.1 | -12.6 | 10.5 | -6.1 | -11.4 | -0.7 |
| Rio de Janeiro | Smoke experimentation | 3.7 | 0.9 | 6.5 | -3.7 | -12.4 | 5.0 | -6.6 | -11.9 | -1.3 |
| Rio de Janeiro | Have been drunk at least once in life | 0.4 | -3.3 | 4.0 | -10.7 | -27.0 | 5.5 | 5.1 | -2.5 | 12.8 |
| Rio de Janeiro | Drugs experimentation | 1.1 | -1.5 | 3.7 | -7.1 | -13.4 | -0.8 | -1.2 | -5.6 | 3.1 |
| Rio de Janeiro | No HPV immunization | -21.1 | -25.2 | -16.9 | -3.6 | -13.1 | 5.9 | -8.4 | -14.7 | -2.2 |
| Rio de Janeiro | Bullying | 5.9 | 2.0 | 9.9 | -1.9 | -6.4 | 2.5 | 0.4 | -5.8 | 6.7 |
| Rio de Janeiro | Thinking life is worthless | 27.1 | 23.7 | 30.4 | -9.1 | -20.3 | 2.1 | -5.5 | -11.6 | 0.7 |
| Rio de Janeiro | Physical violence | 2.3 | -1.3 | 5.9 | -2.0 | -13.8 | 9.9 | -8.3 | -14.2 | -2.4 |
| Rio de Janeiro | No handwashing | 1.3 | -1.7 | 4.3 | -3.3 | -12.2 | 5.6 | -2.7 | -8.7 | 3.2 |
| Rio de Janeiro | Dental pain | 6.0 | 3.0 | 8.9 | 0.9 | -5.9 | 7.8 | -7.0 | -11.9 | -2.0 |
| Rio de Janeiro | Eating disorders | 2.2 | 0.0 | 4.4 | -4.1 | -8.9 | 0.7 | -1.4 | -5.3 | 2.4 |
| São Paulo | UPF (highest quintile) | -1.1 | -3.2 | 1.1 | 0.0 | 0.0 | 0.0 | 3.1 | -2.1 | 8.3 |
| São Paulo | Physical inactivity | 23.0 | 19.1 | 26.9 | 0.0 | 0.0 | 0.0 | 2.4 | -3.8 | 8.5 |
| São Paulo | Smoke experimentation | 2.4 | -2.1 | 6.8 | 0.0 | 0.0 | 0.0 | -11.4 | -17.2 | -5.5 |
| São Paulo | Have been drunk at least once in life | 7.9 | 4.3 | 11.5 | 0.0 | 0.0 | 0.0 | -7.0 | -14.7 | 0.8 |
| São Paulo | Drugs experimentation | 2.2 | -0.7 | 5.0 | 0.0 | 0.0 | 0.0 | -3.4 | -8.5 | 1.7 |
| São Paulo | No HPV immunization | -16.2 | -19.9 | -12.4 | 0.0 | 0.0 | 0.0 | -1.9 | -7.6 | 3.8 |
| São Paulo | Bullying | 11.1 | 7.0 | 15.1 | 0.0 | 0.0 | 0.0 | 2.8 | -3.7 | 9.3 |
| São Paulo | Thinking life is worthless | 28.3 | 25.1 | 31.5 | 0.0 | 0.0 | 0.0 | -5.8 | -12.3 | 0.7 |
| São Paulo | Physical violence | 1.4 | -2.0 | 4.7 | 0.0 | 0.0 | 0.0 | -3.5 | -9.7 | 2.6 |
| São Paulo | No handwashing | 1.0 | -2.1 | 4.2 | 0.0 | 0.0 | 0.0 | -8.8 | -14.9 | -2.7 |
| São Paulo | Dental pain | 5.2 | 1.6 | 8.8 | 0.0 | 0.0 | 0.0 | -5.1 | -10.5 | 0.3 |
| São Paulo | Eating disorders | 2.6 | 0.5 | 4.6 |  |  |  | -5.0 | -8.9 | -1.1 |
| Paraná | UPF (highest quintile) | -1.6 | -5.7 | 2.4 | -12.9 | -17.4 | -8.4 | 5.3 | -1.2 | 11.9 |
| Paraná | Physical inactivity | 20.2 | 15.5 | 24.9 | 16.0 | 6.7 | 25.3 | -5.3 | -13.5 | 2.9 |
| Paraná | Smoke experimentation | 5.5 | 1.3 | 9.7 | 4.1 | -5.7 | 13.9 | -10.6 | -18.7 | -2.5 |
| Paraná | Have been drunk at least once in life | 1.3 | -4.5 | 7.0 | -9.0 | -24.5 | 6.6 | -3.3 | -13.2 | 6.6 |
| Paraná | Drugs experimentation | -1.2 | -4.4 | 1.9 | -11.6 | -18.0 | -5.2 | 2.6 | -3.6 | 8.9 |
| Paraná | No HPV immunization | -10.7 | -14.7 | -6.7 | -10.8 | -17.2 | -4.3 | 5.4 | -1.2 | 12.0 |
| Paraná | Bullying | 5.2 | -0.3 | 10.7 | -2.9 | -14.1 | 8.4 | -8.2 | -16.8 | 0.3 |
| Paraná | Thinking life is worthless | 25.7 | 21.3 | 30.1 | -4.4 | -18.3 | 9.5 | -5.0 | -13.6 | 3.6 |
| Paraná | Physical violence | 1.3 | -2.9 | 5.5 | -7.6 | -18.2 | 3.1 | -2.7 | -10.2 | 4.8 |
| Paraná | No handwashing | -5.5 | -9.6 | -1.4 | -2.2 | -12.2 | 7.8 | 3.3 | -4.3 | 10.9 |
| Paraná | Dental pain | 5.9 | 2.6 | 9.2 | -2.0 | -15.3 | 11.2 | -8.7 | -15.7 | -1.6 |
| Paraná | Eating disorders | 2.8 | -0.1 | 5.7 | -3.8 | -9.9 | 2.2 | 0.5 | -4.3 | 5.3 |
| Santa Catarina | UPF (highest quintile) | -0.3 | -3.3 | 2.7 | -4.8 | -14.4 | 4.9 | 3.0 | -3.3 | 9.2 |
| Santa Catarina | Physical inactivity | 21.2 | 16.7 | 25.7 | 17.8 | 11.9 | 23.6 | -0.2 | -8.0 | 7.5 |
| Santa Catarina | Smoke experimentation | -1.7 | -7.6 | 4.2 | -11.4 | -33.8 | 11.0 | -7.8 | -15.2 | -0.3 |
| Santa Catarina | Have been drunk at least once in life | 1.7 | -3.8 | 7.3 | -9.8 | -25.8 | 6.2 | -2.9 | -12.4 | 6.7 |
| Santa Catarina | Drugs experimentation | 0.8 | -3.3 | 4.8 | -10.8 | -20.7 | -1.0 | 0.0 | -6.3 | 6.2 |
| Santa Catarina | No HPV immunization | -16.3 | -19.8 | -12.8 | -7.0 | -22.5 | 8.6 | -4.6 | -11.4 | 2.1 |
| Santa Catarina | Bullying | 9.1 | 3.8 | 14.3 | 1.2 | -9.5 | 11.8 | -5.8 | -13.9 | 2.3 |
| Santa Catarina | Thinking life is worthless | 26.7 | 22.5 | 31.0 | -7.8 | -22.5 | 6.8 | -15.3 | -23.2 | -7.4 |
| Santa Catarina | Physical violence | 0.9 | -2.8 | 4.6 | 0.8 | -10.9 | 12.6 | -4.2 | -11.3 | 3.0 |
| Santa Catarina | No handwashing | 0.8 | -3.1 | 4.7 | 1.3 | -14.9 | 17.5 | -1.9 | -9.1 | 5.4 |
| Santa Catarina | Dental pain | 8.1 | 4.3 | 11.9 | 3.5 | -3.9 | 11.0 | -8.1 | -14.3 | -1.9 |
| Santa Catarina | Eating disorders | 1.1 | -1.8 | 4.1 | 2.1 | -12.9 | 17.0 | -4.5 | -9.7 | 0.7 |
| Rio Grande do Sul | UPF (highest quintile) | -4.0 | -9.3 | 1.2 | 2.7 | -2.9 | 8.3 | -7.4 | -15.6 | 0.8 |
| Rio Grande do Sul | Physical inactivity | 20.9 | 17.0 | 24.7 | 13.0 | 6.0 | 20.0 | -10.2 | -19.2 | -1.2 |
| Rio Grande do Sul | Smoke experimentation | 4.3 | -1.7 | 10.4 | 14.7 | 0.7 | 28.8 | -5.0 | -14.0 | 3.9 |
| Rio Grande do Sul | Have been drunk at least once in life | 2.0 | -5.9 | 10.0 | 6.3 | -7.7 | 20.4 | -6.7 | -18.1 | 4.7 |
| Rio Grande do Sul | Drugs experimentation | 3.9 | -1.5 | 9.2 | -4.9 | -9.9 | 0.1 | -1.3 | -8.5 | 6.0 |
| Rio Grande do Sul | No HPV immunization | -19.3 | -25.3 | -13.4 | -2.3 | -21.6 | 17.1 | -4.6 | -12.4 | 3.3 |
| Rio Grande do Sul | Bullying | 7.6 | 2.8 | 12.4 | 4.0 | -11.5 | 19.6 | -4.8 | -14.4 | 4.9 |
| Rio Grande do Sul | Thinking life is worthless | 29.0 | 24.8 | 33.2 | -8.4 | -23.2 | 6.5 | -18.3 | -27.7 | -8.9 |
| Rio Grande do Sul | Physical violence | -1.1 | -4.5 | 2.3 | -1.5 | -5.5 | 2.6 | -9.1 | -17.6 | -0.5 |
| Rio Grande do Sul | No handwashing | -2.5 | -6.2 | 1.2 | -6.0 | -11.9 | -0.1 | 6.1 | -1.7 | 13.8 |
| Rio Grande do Sul | Dental pain | 3.6 | -1.1 | 8.4 | 1.4 | -4.9 | 7.8 | -9.9 | -17.8 | -2.0 |
| Rio Grande do Sul | Eating disorders | 3.6 | 0.9 | 6.3 | 0.8 | -1.5 | 3.1 | -2.1 | -6.9 | 2.8 |
| Mato Grosso do Sul | UPF (highest quintile) | 1.0 | -1.1 | 3.1 | -0.2 | -7.6 | 7.1 | -1.2 | -6.6 | 4.3 |
| Mato Grosso do Sul | Physical inactivity | 19.2 | 15.1 | 23.4 | 3.8 | -1.4 | 9.0 | -2.1 | -9.4 | 5.3 |
| Mato Grosso do Sul | Smoke experimentation | 3.5 | 0.0 | 7.0 | -12.5 | -19.8 | -5.3 | -9.2 | -16.4 | -2.0 |
| Mato Grosso do Sul | Have been drunk at least once in life | 4.1 | -0.1 | 8.4 | -12.0 | -21.0 | -3.0 | -3.9 | -13.3 | 5.6 |
| Mato Grosso do Sul | Drugs experimentation | 0.5 | -2.5 | 3.6 | -9.2 | -13.3 | -5.2 | -3.9 | -9.4 | 1.6 |
| Mato Grosso do Sul | No HPV immunization | -16.4 | -22.1 | -10.7 | -3.8 | -10.3 | 2.6 | -4.0 | -11.7 | 3.6 |
| Mato Grosso do Sul | Bullying | 6.4 | 2.9 | 9.9 | 14.7 | 4.8 | 24.6 | -4.8 | -12.5 | 2.8 |
| Mato Grosso do Sul | Thinking life is worthless | 26.1 | 20.8 | 31.4 | -7.9 | -15.4 | -0.3 | -2.3 | -9.8 | 5.3 |
| Mato Grosso do Sul | Physical violence | 5.7 | 3.1 | 8.3 | -2.4 | -14.9 | 10.2 | -2.8 | -9.9 | 4.3 |
| Mato Grosso do Sul | No handwashing | 0.7 | -3.0 | 4.4 | -4.9 | -10.9 | 1.2 | 1.4 | -5.3 | 8.0 |
| Mato Grosso do Sul | Dental pain | 5.0 | 1.9 | 8.1 | -2.9 | -6.3 | 0.5 | -10.4 | -16.5 | -4.3 |
| Mato Grosso do Sul | Eating disorders | 3.9 | 1.3 | 6.4 | 9.5 | -6.0 | 25.0 | -3.1 | -8.0 | 1.7 |
| Mato Grosso | UPF (highest quintile) | -1.3 | -4.3 | 1.7 | 0.6 | -13.2 | 14.4 | 9.2 | 2.7 | 15.8 |
| Mato Grosso | Physical inactivity | 16.3 | 11.9 | 20.7 | 15.2 | 8.5 | 22.0 | -4.6 | -12.9 | 3.8 |
| Mato Grosso | Smoke experimentation | -6.6 | -11.7 | -1.6 | 4.4 | -11.8 | 20.6 | -7.1 | -15.4 | 1.3 |
| Mato Grosso | Have been drunk at least once in life | 4.0 | -1.4 | 9.4 | 9.4 | -4.1 | 22.8 | -9.4 | -20.8 | 2.0 |
| Mato Grosso | Drugs experimentation | -0.7 | -3.4 | 1.9 | -2.5 | -12.0 | 6.9 | -2.7 | -8.8 | 3.4 |
| Mato Grosso | No HPV immunization | -16.6 | -23.2 | -10.0 | -2.5 | -10.6 | 5.7 | -1.9 | -10.9 | 7.0 |
| Mato Grosso | Bullying | 10.5 | 4.5 | 16.6 | -1.3 | -11.3 | 8.7 | -1.7 | -10.9 | 7.6 |
| Mato Grosso | Thinking life is worthless | 25.7 | 18.8 | 32.5 | -9.7 | -16.8 | -2.7 | -3.7 | -13.0 | 5.5 |
| Mato Grosso | Physical violence | 4.0 | 0.6 | 7.4 | -18.4 | -23.4 | -13.4 | 5.2 | -2.6 | 13.0 |
| Mato Grosso | No handwashing | -2.6 | -7.6 | 2.4 | -6.5 | -16.7 | 3.6 | 5.9 | -2.1 | 13.9 |
| Mato Grosso | Dental pain | 4.8 | 0.1 | 9.4 | -0.3 | -8.4 | 7.9 | -6.6 | -14.4 | 1.2 |
| Mato Grosso | Eating disorders | 2.2 | -0.2 | 4.6 | -3.3 | -7.9 | 1.3 | 5.2 | -0.3 | 10.6 |
| Goiás | UPF (highest quintile) | -1.5 | -3.8 | 0.8 | -6.5 | -10.3 | -2.7 | 10.4 | 6.1 | 14.7 |
| Goiás | Physical inactivity | 21.7 | 19.4 | 24.1 | 16.7 | 5.5 | 27.8 | 0.0 | -5.7 | 5.6 |
| Goiás | Smoke experimentation | -3.2 | -6.5 | 0.2 | 1.0 | -4.4 | 6.5 | -5.6 | -11.0 | -0.2 |
| Goiás | Have been drunk at least once in life | -0.7 | -5.5 | 4.2 | 5.0 | -3.6 | 13.5 | 0.2 | -7.4 | 7.8 |
| Goiás | Drugs experimentation | -1.7 | -3.7 | 0.4 | -6.3 | -10.7 | -1.9 | -0.9 | -5.1 | 3.3 |
| Goiás | No HPV immunization | -20.2 | -24.5 | -16.0 | 3.5 | -18.1 | 25.1 | -6.4 | -12.2 | -0.6 |
| Goiás | Bullying | 8.9 | 5.2 | 12.6 | -3.2 | -11.7 | 5.2 | -0.8 | -6.9 | 5.4 |
| Goiás | Thinking life is worthless | 26.5 | 23.1 | 29.9 | -12.0 | -24.6 | 0.6 | -7.2 | -13.3 | -1.2 |
| Goiás | Physical violence | 1.2 | -1.5 | 4.0 | -16.6 | -22.2 | -11.0 | 1.5 | -3.9 | 6.9 |
| Goiás | No handwashing | 1.2 | -2.0 | 4.3 | -2.1 | -8.3 | 4.2 | -2.1 | -7.4 | 3.2 |
| Goiás | Dental pain | 7.1 | 4.1 | 10.2 | 7.0 | -10.2 | 24.1 | -6.6 | -11.5 | -1.6 |
| Goiás | Eating disorders | 2.7 | 1.1 | 4.3 | -5.4 | -10.8 | 0.1 | -0.9 | -4.5 | 2.7 |
| Distrito Federal | UPF (highest quintile) | -1.2 | -5.3 | 2.9 | 6.7 | 4.8 | 8.6 | -1.9 | -7.7 | 4.0 |
| Distrito Federal | Physical inactivity | 24.7 | 21.5 | 28.0 | -10.0 | -12.5 | -7.5 | -3.4 | -10.3 | 3.6 |
| Distrito Federal | Smoke experimentation | 0.3 | -4.5 | 5.2 | -3.4 | -7.3 | 0.6 | -15.1 | -21.9 | -8.4 |
| Distrito Federal | Have been drunk at least once in life | 7.0 | 0.1 | 13.9 | -4.1 | -7.1 | -1.1 | -6.5 | -16.1 | 3.0 |
| Distrito Federal | Drugs experimentation | 3.7 | -0.2 | 7.6 | -5.8 | -9.5 | -2.1 | -1.7 | -7.8 | 4.5 |
| Distrito Federal | No HPV immunization | -21.9 | -26.3 | -17.5 | -4.6 | -6.5 | -2.6 | -3.2 | -10.2 | 3.8 |
| Distrito Federal | Bullying | 5.8 | -0.4 | 12.0 | 6.5 | 2.9 | 10.1 | -8.6 | -16.2 | -1.0 |
| Distrito Federal | Thinking life is worthless | 26.5 | 22.8 | 30.3 | -3.8 | -6.9 | -0.8 | -15.3 | -22.8 | -7.8 |
| Distrito Federal | Physical violence | 0.3 | -4.6 | 5.2 | 3.5 | 0.3 | 6.7 | -7.6 | -14.5 | -0.8 |
| Distrito Federal | No handwashing | 3.1 | -2.5 | 8.7 | -16.0 | -18.1 | -13.9 | -3.6 | -10.8 | 3.6 |
| Distrito Federal | Dental pain | 6.7 | 3.1 | 10.4 | 6.3 | 4.2 | 8.5 | -4.3 | -10.0 | 1.5 |
| Distrito Federal | Eating disorders | 3.7 | 1.5 | 5.9 | 6.1 | 4.7 | 7.5 | 1.2 | -3.0 | 5.3 |

*Gender difference: Negative values indicate higher prevalence among boys, positive differences indicate higher prevalence among girls*

*Area difference: Negative values indicate higher prevalence among urban residents, positive differences indicate higher prevalence among rural residents*

*Wealth SII: Negative values indicate higher prevalence among the poorest, positive differences indicate higher prevalence among the wealthiest*
